# Plant endemism of the Balkan high‐mountain fens: a community‐level synthesis and their climate change perspective

**DOI:** 10.1111/plb.70279

**Published:** 2026-08-16

**Authors:** M. Hájek, P. Hájková, M. Chobanova, Z. Plesková, E. Bergmeier, P. Lazarević, D. Szokala, N. Berisha, L. Shuka, D. Dítě, T. Peterka, R. Čusterevska, D. Sopotlieva, J. Divíšek, I. Goia, B. Vrábel, Đ. Milanović, S. Hristovski, D. Stešević, P. Bureš, I. Apostolova

**Affiliations:** ^1^ Department of Botany and Zoology, Faculty of Science Masaryk University Brno Czech Republic; ^2^ Department of Paleoecology Institute of Botany, The Czech Academy of Sciences Brno Czech Republic; ^3^ Institute of Biology, Faculty of Natural Sciences and Mathematics Ss. Cyril and Methodius University in Skopje Skopje North Macedonia; ^4^ Department of Vegetation Analysis and Phytodiversity, Albrecht‐von‐Haller Institute of Plant Sciences University of Göttingen Göttingen Germany; ^5^ Institute of Botany and Botanical Garden Jevremovac, Faculty of Biology University of Belgrade Belgrade Serbia; ^6^ Department of Biology, Faculty of Mathematics and Natural Sciences University of Prishtina Prishtina Kosovo; ^7^ Department of Biology, Faculty of Natural Sciences University of Tirana Tirana Albania; ^8^ Plant Science and Biodiversity Center Slovak Academy of Sciences Bratislava Slovakia; ^9^ Institute of Biodiversity and Ecosystem Research Bulgarian Academy of Sciences Sofia Bulgaria; ^10^ Department of Taxonomy and Ecology, Faculty of Biology and Geology, Centre for Systems Biology, Biodiversity and Bioresources (3B) Babeş‐Bolyai University Cluj‐Napoca Romania; ^11^ Faculty of Forestry University of Banja Luka Banja Luka Bosnia and Herzegovina; ^12^ Faculty of Natural Sciences and Mathematics University of Montenegro Podgorica Montenegro

**Keywords:** Biogeography, diversity, habitat islands, palaeoclimate, specialists, wetland

## Abstract

- The phenomenon of Balkan high‐mountain fen plant endemism, unique within Europe, remains poorly studied. We compiled an original dataset of wetland vegetation‐plot data across nine countries where Balkan endemics and subendemics occur. We tested the diversity patterns for endemics, non‐endemic habitat specialists of bryophytes and vascular plants and matrix‐derived taxa with respect to climate, palaeoclimate, edaphic and topographic factors.
- We used Spearman correlations, Spatial Autoregressive Error Models, Canonical Correspondence Analysis and Principal Component Analysis of abiotic factors with richness patterns projected using Generalised Additive Models.
- Two major hotspots of fen endemism, centred around the Rila and Korab Mts, were identified. Species composition of endemics generally follows an east‐to‐west gradient that coincides with palaeoclimatic patterns. Palaeoclimate was influential for community‐scale richness of bryophytes and narrow‐range endemics, the latter being positively affected by the Bølling–Allerød precipitation peak and the wet‐tundra climate during the glacial. The richness of other groups was affected by recent climate, edaphic factors and topography. Water pH differentiated major vegetation types and affected the richness of matrix‐derived taxa and fen bryophytes, but had a minor impact on Balkan endemics, except those associated with serpentinite. Several endemic taxa usually co‐exist even in small wetland patches.
- Palaeoclimate shows that fen endemics evidently experienced drier, warmer summers than today over the last 21,000 years; yet they were more affected by past precipitation. If we conserve Balkan high‐mountain fens, their catchment properties and hydrology, and maintain moderate grazing, we increase the likelihood that their endemics will withstand ongoing climate change.

## Graphical abstract

Balkan high‐mountain fens harbour unique endemic plant communities, with a west‐to‐east compositional gradient and local richness primarily shaped by palaeoclimate. As these fens have already experienced drier, warmer summers than at present, they may withstand climate change if actively and effectively protected.

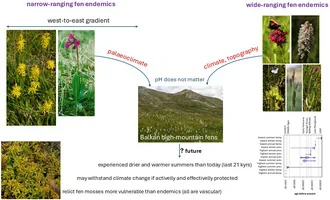

## INTRODUCTION

Narrow endemism refers to taxa restricted to a small area such as an island, archipelago, or one or several neighbouring mountain ranges (Myers *et al*. 2000). The narrow‐range endemics deserve increased attention, as they inherently serve as objects of conservation efforts, being an important part of the national or regional natural heritage and stewardship commitment. Apart from their rarity, endemics are attracting scientific interest because they can tell compelling stories about evolutionary and natural history. They may originate from the disjunct populations of either once widespread taxa whose distribution range had fragmented (Myers *et al*. 2000), or from ancestral populations that colonised long‐isolated islands and evolved independently (Whittaker *et al*. 2017). High mountains serve as centres of endemism worldwide (Cai *et al*. 2023; Love *et al*. 2023; Ronikier *et al*. 2023). They usually harbour habitats with long‐term cool conditions, surrounded by an ‘ocean’ of warmer climate that supports contrasting ecosystems. North of the equator, mountain summits and basins also acted as coldland refugia during the warmer periods of the Quaternary, thereby supporting disjunct southern populations of boreal or arctic–alpine taxa (Birks & Willis 2008; Stewart *et al*. 2010). Frequent Quaternary climate oscillations repeatedly connected otherwise disjunct populations through the expansion and contraction of cold‐adapted habitats. In addition, repeated glaciation of the highest elevations forced populations to relocate in many areas, especially in the northern latitudes (Birks & Willis 2008; Stewart *et al*. 2010; Love *et al*. 2023). Besides promoting extinctions, this spatio‐temporal dynamic prevents speciation into narrow‐range endemics, particularly in habitats that are rare within the landscape mosaic and form ‘islands within islands’, such as mires. As a result, mountain mires tend to exhibit low levels of endemism—particularly in Europe, where environmental conditions fluctuated markedly throughout the Quaternary and especially after the Last Glacial Maximum (LGM) (Birks & Willis 2008; Hughes *et al*. 2022). Fen endemism is relatively rare in North America (Jules *et al*. 2011) and especially in Europe (Hobohm & Bruchmann 2011), in contrast to Middle Asia (Naqinezhad *et al*. 2021; Nowak *et al*. 2025). In Central and Southern Europe, sporadic occurrences of non‐endemic coldland taxa in mires instead represent disjunct relicts of boreo‐continental or arctic–alpine taxa (Strid 1993; Apostolova *et al*. 2013; Vukojičić *et al*. 2014; Dítě *et al*. 2018; Kougioumoutzis *et al*. 2021). In Central Europe, the rare examples represent fen neoendemics with unclear taxonomic position such as *Pedicularis sudetica* Willd., *Carex derelicta* Štěpánková or *Dactylorhiza maculata* subsp. *sudetica* (Poech ex Rchb. f.) Vöth. These taxa form only a minute proportion in their regional species pool. In contrast, in southern Europe, narrow‐range endemics often occur in fens and form a substantial component of plant communities in the Sierra Nevada (Rivas‐Martínez *et al*. 2002), Corsica (Gamisans 1972) and the Balkans (Quézel 1964; Lakušić 1968; Lakušić & Grgić 1971; Roussakova 2000), assembling distinct vegetation types (Lakušić 1968; Gamisans 1972; Rivas‐Martínez *et al*. 2002; Peterka *et al*. 2017). These findings led to the recognition of ‘Relict mires of Mediterranean mountains’ as a distinct habitat in the pan‐European habitat classification system EUNIS (Chytrý *et al*. 2020). In the European Red List of Habitats (Janssen *et al*. 2016), this habitat is categorised as Vulnerable, although data from the Balkans were under‐represented. Given the accelerating climate change and summer heatwaves threatening isolated coldland ecosystems (Burić 2024; Shumka *et al*. 2024), greater attention should be given to these unique, endemic‐rich fens.

Quaternary climate stability supports endemism (Weigelt *et al*. 2016; Sandel *et al*. 2017). This general pattern may also apply to the southern European mountains, where a more stable palaeoclimate might positively affect endemism, but this has not yet been tested. It also remains unclear how the effects of climate and palaeoclimate patterns manifest at the community level. The presence of several specialist or endemic species co‐occurring in local communities may indicate their long regional history and well‐developed inter‐specific interactions, which together promote long‐term survival and resilience to environmental changes (Verdú & Valiente‐Banuet 2008). Identifying community‐level diversity hotspots of endemics is therefore essential for conservation planning.

Here we focus on the Balkans and the adjacent high mountains of the Southern Carpathians, which share a common pool of Balkan endemic and subendemic taxa (hereafter referred to as *endemics*) occurring in fens, a habitat that is generally small and highly fragmented in this region (Tanneberger *et al*. 2017; Chobanova *et al*. 2025). The study mountain area spans over nine countries and covers approximately 100,000 km^2^ when calculated as the area above 1,000 m a.s.l. (J.D., unpublished data). Some endemic taxa are wide‐ranging, distributed across the high mountains of the entire Balkans and may even extend slightly beyond them. Others are narrow‐ranging, restricted to smaller areas, reflecting limited dispersal ability and a stronger dependence on specific environmental, contemporary or historical conditions (Stevanović *et al*. 2007; Petrova & Vladimirov 2010).

Although the high mountains of the Balkans are significant hotspots of high‐mountain endemism (Stevanović *et al*. 2007; Ronikier *et al*. 2023), they are often neglected in global syntheses and reviews (*e.g*. Love *et al*. 2023). Moreover, no synthetic study has focused on endemism in mires, which, although less developed than in drier mountain habitats, is exceptional within the boreal Holarctic region. In the high mountains of the Balkans and Southern Carpathians, where ombrotrophic bogs are missing (Chytrý *et al*. 2020), mire habitats are represented by fens including transitions to springs. Only local or regional checklists and vegetation overviews have addressed the phenomenon of fen endemism (Horvat *et al*. 1974; Petrova & Vladimirov 2010; Tomović *et al*. 2014; Peterka *et al*. 2017). The disjunct populations of boreal or arctic–alpine taxa have attracted more attention (Hájek *et al*. 2009; Apostolova *et al*. 2013; Vukojičić *et al*. 2014; Kougioumoutzis *et al*. 2021; Bergmeier 2023). Nevertheless, Hájková *et al*. (2006) found that diversity patterns along environmental gradients in the Balkan high mountains differ among boreal, arctic–alpine and narrower range alpine taxa. Balkan endemics with an ecological optimum in fens have also been neglected in taxonomic and phylogeographic studies, compared to taxa of tundra, scree or rock habitats (Španiel & Rešetnik 2022; Ronikier *et al*. 2023). No study has comprehensively addressed high‐mountain endemics across the entire Balkans and their co‐existence in fen communities, or the drivers of local diversity and the role of Quaternary climate dynamics. Using complete plot records of vascular plants and bryophytes, together with edaphic and macroclimatic data, we aimed to (i) critically review the list of Balkan endemics associated with spring and fen plant communities, (ii) identify major hotspots of fen endemism in the Balkans, (iii) associate individual endemic taxa with specific regions and vegetation types and (iv) test the response of the local diversity of wide‐ and narrow‐range endemics and other groups of taxa to the edaphic, climatic and palaeoclimatic variables.

## MATERIAL AND METHODS

### Study area and its climate

The study area comprises the high mountains of the Balkan Peninsula and the adjacent Southern Carpathians (Romania). The area is shared by nine countries: Albania, Bosnia and Herzegovina, Bulgaria, Greece, Kosovo, Montenegro, North Macedonia, Romania and Serbia. It includes major mountain ranges of southeastern Europe, such as the Southern Carpathians, the Central Balkan range, the Rila‐Rhodope massif, the Scardo‐Pindus range, the Dinarids and others (Fig. 1). Only those parts of these ranges where high‐mountain fens with Balkan endemics occur were included. From a plant‐geographical perspective, the region belongs to the Illyrian and Balkanic regions (divided into West‐Moesian, East‐Moesian and Scardo‐Pindic subregions) (Horvat *et al*. 1974). The individual parts of the study area differ in current climate, palaeoclimate, topography and edaphic factors, the effects of which were tested in this study. The high‐resolution climate map of Botti (2018) shows that Balkan high mountains represent isolated cold‐climate islands. According to the updated Köppen climate classification (Beck *et al*. 2023), most of these mountain islands belong to the Dfb zone (cold with no dry season and warm summers), but the southern and southeastern parts of the study area transition to or belong to the Dsb zone (cold with dry warm summer). During the Quaternary, isolated mountain glaciers existed during the LGM between 29,000 and 19,000 BP (Kuhlemann *et al*. 2009; Tonkov 2021; Hughes *et al*. 2022). Between them, alpine tundra locally developed (Tonkov 2021). The mountain steppe was the dominant habitat throughout the glacial periods, as indicated by Artemisia‐, Poaceae‐, Ephedra‐ and Juniperus‐type pollen, together with additional grassland indicators (Tonkov 2021). The relatively dry climate of the Balkan high mountains persisted throughout the Late Pleniglacial (Late High Glacial) and the early Holocene (Wright Jr *et al*. 2003). According to Divíšek *et al*.  (2022), the Balkans were among the climatically most suitable areas for steppes in Europe during both the LGM and the Late Pleniglacial. This late Pleistocene climate history supports the hypothesis that fen endemism is higher in regions that have continuously retained tundra conditions, at least locally, within landscapes otherwise dominated by dry steppe or forest‐supporting climates. Considering the magnitude of climate variability from the LGM to the present, which well represents Quaternary climate variability (Sandel *et al*. 2017), our focus on the last 21,000 years may provide reliable insights into the past drivers of fen endemism.

**Fig. 1:**
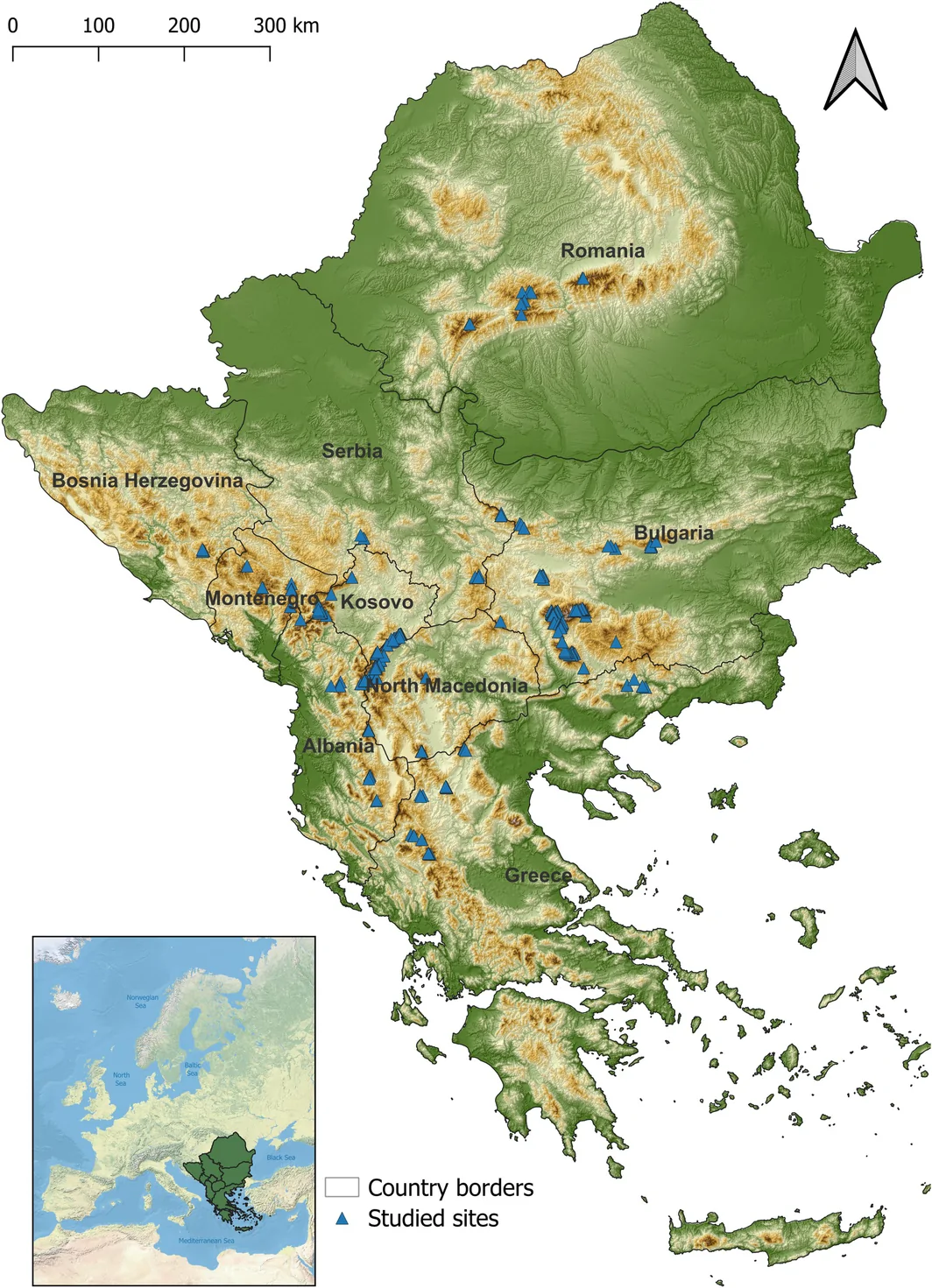
The distribution of the relevés included in the analyses.

### The list of Balkan endemics, selection of vegetation‐plot records and taxonomic harmonisation

As the first step, we assembled the candidate list of Balkan endemics whose occurrence is at least partially associated with fens (Table S1, see Fig. S1 for photos). The plant nomenclature was harmonised using Euro+Med PlantBase (Euro+Med 2006), with a few exceptions described in Table S1. As next steps, described in detail in Appendix S1, we reduced the list and selected the vegetation‐plot records (referred to as relevés) for analysis. The resulting pool of relevés comprises our original data and a limited number of relevés from other authors (Table S2). The dataset was assembled during field research focused on sedge‐moss spring and fen vegetation; other vegetation types, such as matgrass pasture and wet heath, were omitted. The applied selection rule resulted in 410 selected relevés, out of which 376 relevés contained species composition of the moss layer. The taxa were classified into narrow‐range endemics (7 taxa), wide‐range endemics (18 taxa), vascular plants specialised to fens (57 taxa), bryophytes specialised to fens (53 taxa) and other taxa (415), following the assignments of Peterka *et al*. (2017), Horsáková *et al*. (2018) and Hájek *et al*. (2022). See Table S1 for species lists, Appendix S1 for how taxonomic uncertainties and equivocal categorisations were addressed and for the list of mountain ranges included in the resulting dataset.

Vegetation plot data were collected using the Braun‐Blanquet approach with the nine‐grade cover scale (Westhoff & van der Maarel 1978) during the summer months (June to August) from 2001 to 2025 across all elevations, but the analysed dataset was restricted during data filtering, resulting in a minimum elevation of approximately 1000 m (Appendix S1). Both the original nine‐grade scale and its conversion to mid‐point values (according to the JUICE software, Tichý 2002) were used in the individual analyses. Although most plots were sampled using the same protocol with a standard plot size of 16 m^2^, the analysed dataset includes plots of different sizes (2–100 m^2^). Smaller plot sizes were used when the spring or the fen containing Balkan endemics occurred only fragmentarily, which prevented larger plot sizes. Larger plot sizes primarily come from the database or unpublished data of L. Melovski. Plot size was included as a predictor in subsequent analyses. The motivation for treating plot size as an independent factor was to test for potential differences in the plot‐size effect across species groups.

### Environmental variables

We supplemented individual relevés with the direct field measurements of water pH and conductivity (EC; μS.cm^−1^). For the 132 and 147 relevés, respectively (primarily data from external authors), we imputed missing values using the Moss method (Tichý *et al*. 2010). To account for their synergistic or mutually substitutive effects, water pH and EC were combined into a single variable (adjusted pH) as described by Plesková *et al*. (2016). To control for potential differences in hydrology, we also calculated the arithmetic mean of ecological indicator values (EIV) for water level, weighted by inverse species tolerances (niche width), using European‐scale values for both vascular plants and bryophytes (Hájek *et al*. 2020).

We further extracted, for the location of each relevé, the current and the past macroclimate parameters including mean annual temperature (bio1), mean temperature of warmest quarter (bio10; summer temperature), mean temperature of the coldest quarter (bio11; winter temperature), annual precipitation (bio12), precipitation of warmest quarter (bio18; summer precipitation) and precipitation of coldest quarter (bio19; winter precipitation). These variables were obtained at a spatial resolution of 30 arc seconds from the CHELSA Timeseries and CHELSAcruts datasets as 50‐year averages for 1940–1989 AD (Karger *et al*. 2017), and from downscaled and debiased palaeoclimate simulations of the Community Climate System Model ver. 3 (CCSM3; Divíšek 2021) for past periods. Based on palaeoclimate data spanning the last 21,000 years at a 100‐year temporal resolution, we calculated the maximum positive and negative deviations (absolute values) from the recent value for each of the above‐mentioned variables (see picture in Appendix S1 for an example) and the climate stability index. For precipitation variables, the deviation was expressed as a percentage of the recent value. The index of climate stability was calculated as follows: for bio10 and bio11, an average absolute deviation from the recent value was calculated. For bio18 and bio19, we calculated the average percentage deviation from the recent value. Following Herrando‐Moraira *et al*. (2022), each of these deviations was then normalised to values between 0 and 1, with 0 representing completely stable areas and 1 the most unstable ones. Overall climate stability since the LGM was then calculated for each relevé as 1 minus the mean of the normalised deviations. Higher values of the index thus indicate higher climate stability.

We further used a digital elevation model at a 90 m resolution (Jarvis *et al*. 2008) and calculated elevation (m a.s.l.) and the Terrain Ruggedness Index (TRI) for each site. We also created 5 km and 10 km buffer zones around each relevé, and within these zones, we calculated minimum, mean and maximum elevation and TRI, as well as elevation and TRI ranges. Finally, within these buffer zones, we also calculated the area at elevations above 1,700 m, with the premise that a larger area indicates a more massive mountain range, buffering the effects of short‐term climate fluctuations and, hence, showing more stable cold‐climate conditions recently and more opportunities to find cold‐microclimate refugia during any warm period.

### Mapping the hotspots

We identified hotspots of community‐scale diversity in Balkan fen endemics by applying kernel density estimation (KDE) to point‐based relevé data using the Heatmap (KDE) tool in QGIS 3.34 (QGIS.org 2026). A fixed search radius (bandwidth) of 50 km was applied uniformly across all plant groups to ensure comparability of spatial patterns. This relatively wide radius was chosen to produce generalised results. Each relevé was weighted by the number of taxa within the respective plant group, using the corresponding attribute field as a weight parameter in the QGIS Heatmap tool. The output was visualised using a continuous colour ramp representing gradients from low to high diversity, with each panel displayed using an independent colour scale.

### Data analyses

Vegetation was classified into broad types using the modified TWINSPAN algorithm (Roleček *et al*. 2009), which, unlike the original TWINSPAN (Hill *et al*. 1975), divides only the most heterogeneous cluster from the second division onwards, as indicated by Whittaker's beta diversity measure in this study. The algorithm was run on presence/absence data, with cover‐abundance values partially retained by cloning the taxon if its cover value exceeded the threshold (*pseudo‐species cut‐levels* according to Hill *et al*. 1975). Thresholds of 5% (2m code) and 25% (3) were adopted, above which the taxon was doubled or tripled, respectively. Divisions were stopped at four clusters. For justification of the method selection and stopping rule, see Appendix S1. The diagnostic taxa for the clusters were delimited by the combination of the Fisher's exact test, phi‐coefficient and frequency; see Fig. S2 for thresholds.

Only 376 relevés with identified bryophytes were included. For the 375 relevés (see Table S2 for an explanation of why one relevé was excluded), the complex space of 32 environmental variables was reduced to two dimensions using Principal Component Analysis (PCA; see Appendix S1).

The effects of individual explanatory variables on the species composition of Balkan endemics (both groups merged; *Cirsium epiroticum* Petr. excluded due to outlier effects) at the community level were tested using Canonical Correspondence Analysis (CCA) with a Monte Carlo test. The mid‐point percentage values were log‐transformed. Forward selection was stopped when the contribution of a newly added variable fell below 5%, as more liberal thresholds produced a strong arch effect.

For each group (narrow‐range endemics, wide‐range endemics, all endemics, non‐endemic fen vascular plants, non‐endemic fen bryophytes and other, *i.e*. matrix‐derived taxa), we tested the Spearman rank correlations between richness and environmental variables. Resulting *p*‐values were adjusted using the Bonferroni correction to address multiple testing. We then tested how the results change when controlled for multicollinearity and spatial autocorrelation using the spatial simultaneous autoregressive error model (SAR_err_). For SAR_err_, we selected environmental variables that significantly correlated with any richness value. If the Spearman correlation between any two selected variables exceeded 0.7, we removed the variable with the lower correlation with taxa richness. The spatial weight matrix used by SAR_err_ was constructed from Moran's correlograms calculated for each group's taxonomic richness using the *correlog* function in the *pgirmess* R package. The distance at which spatial autocorrelation decreased to zero was recorded, and plots within this distance were considered neighbours when constructing the spatial weight matrix. We interpreted the results of both Spearman correlations and SAR_err_ in the light of the fact that climate, especially palaeoclimate variables modelled at coarse spatial scales, is inherently strongly spatially autocorrelated, leading to penalisation in the SAR_err_ models.

We further classified individual relevés by their location within 11 predefined regions (Table S2), for which we calculated the percentage frequency of each Balkan endemic taxon. The resulting taxon‐by‐region matrix was subjected to Principal Coordinate Analysis (PCoA) using the Hellinger distance as a dissimilarity measure. Note that the distance is applied to non‐transformed compositional proportions (percentage frequencies), depending on the regional boundaries defined in Table S2.

PCA, CCA and PCoA were analysed using Canoco 5 software (ter Braak & Šmilauer 2012). TWINSPAN and diagnostic taxa delimitation were performed in JUICE 7.1.25 software (Tichý 2002). Other statistical analyses were conducted using the R 4.2.1 package (R Core Team 2022).

## RESULTS

The vast majority (84%) of the 410 relevés that met the inclusion criteria (*i.e*. belonging to a fen or spring habitat and containing at least one endemic taxon from the list) were sampled during the targeted field research conducted by the authors. The relevé dataset contained 550 plant taxa. The dataset is geographically well balanced between the east and the west; the number of relevés is roughly the same east and west of 21.55° E, dividing the area approximately into two halves, each spanning approximately 300 km eastwards or westwards (Fig. 1).

### Pattern across vegetation types

The hierarchical classification (TWINSPAN) first divided the relevé dataset into calcareous springs and fens, and acidic *Sphagnum* fens. According to the phi coefficient and Fisher's exact test, the former are most strongly characterised by *Palustriella commutata* (Hedw.) Ochyra, *Ptychostomum pseudotriquetrum* (Hedw.) J. R. Spence & H. P. Ramsay ex Holyoak & N. Pedersen, *Blysmus compressus* (L.) Link and *Juncus alpinoarticulatus* Chaix, while the latter are characterised by *Sphagnum subsecundum* Nees, *Warnstorfia exannulata* (Schimp.) Loeske and *Carex echinata* Murray (Fig. S2). The former (calcicole) group was then divided into the two clusters differing by representation of individual endemic taxa (*Narthecium scardicum* Košanin, *Carex castroviejoi* Luceño & Jim.‐Mejías and *Pinguicula hirtiflora* Ten. *versus Willemetia stipitata* subsp. *albanica* (Kümmerle & Jáv.) Kirschnerová, *Cardamine acris* Griseb., *Silene asterias* Griseb. and *Juncus thomasii* Ten.). The second (acidic) group was divided into a more widely distributed cluster characterised by widespread taxa of poor and moderately rich fens including some endemics (*Cirsium heterotrichum* Pančić, *Epilobium vernonicum* Snogerup and *Crocus veluchensis* Herb.) and the cluster characterised among others by the narrow‐range Balkan endemics *Primula frondosa* subsp. *exigua* (Velen.) O. Schwarz, *Primula deorum* Velen. and some others (Fig. S2). See also Table S3 for a full synoptic table and Fig. S3 for photos illustrating the vegetation.

### Identifying the hotspots

We found the highest concentration of Balkan fen endemic taxa at the community level in the south‐western part of the Rila‐Rhodope mountain system (the Rila, Pirin and Vitosha Mts) and in the mountain node between North Macedonia, Albania and Kosovo (Šar and Korab Mts) (Fig. 2d,f). These areas show both the highest mean number of endemics per relevé (max. Vitosha Mts, 4.7 taxa) and the highest individual maxima in Korab Mts (Crn Kamen site, 9 taxa) and the Rila Mts (Seven Lakes site, 8 taxa) (Table S4). The same pattern appeared for fen vascular plants and matrix‐derived taxa (Fig. 2b,e). When focusing only on the narrow‐range endemics, the primary hotspot was identified in the Rila Mts in Bulgaria, while the serpentinite mountains of the southwestern Balkans (East Albania, NW Greece) also appeared important (Fig. 2c, Table S4). The pattern for fen specialists of bryophytes, however, differed. They show a primary hotspot in the Rila Mts (Fig. 2a), as in the case of narrow‐ranged endemics, but shows multiple secondary hotspots (Pirin, Šar, Vitosha Mts) that also include the Southern Carpathians (the Lotrului and Cindrel Mts).

**Fig. 2:**
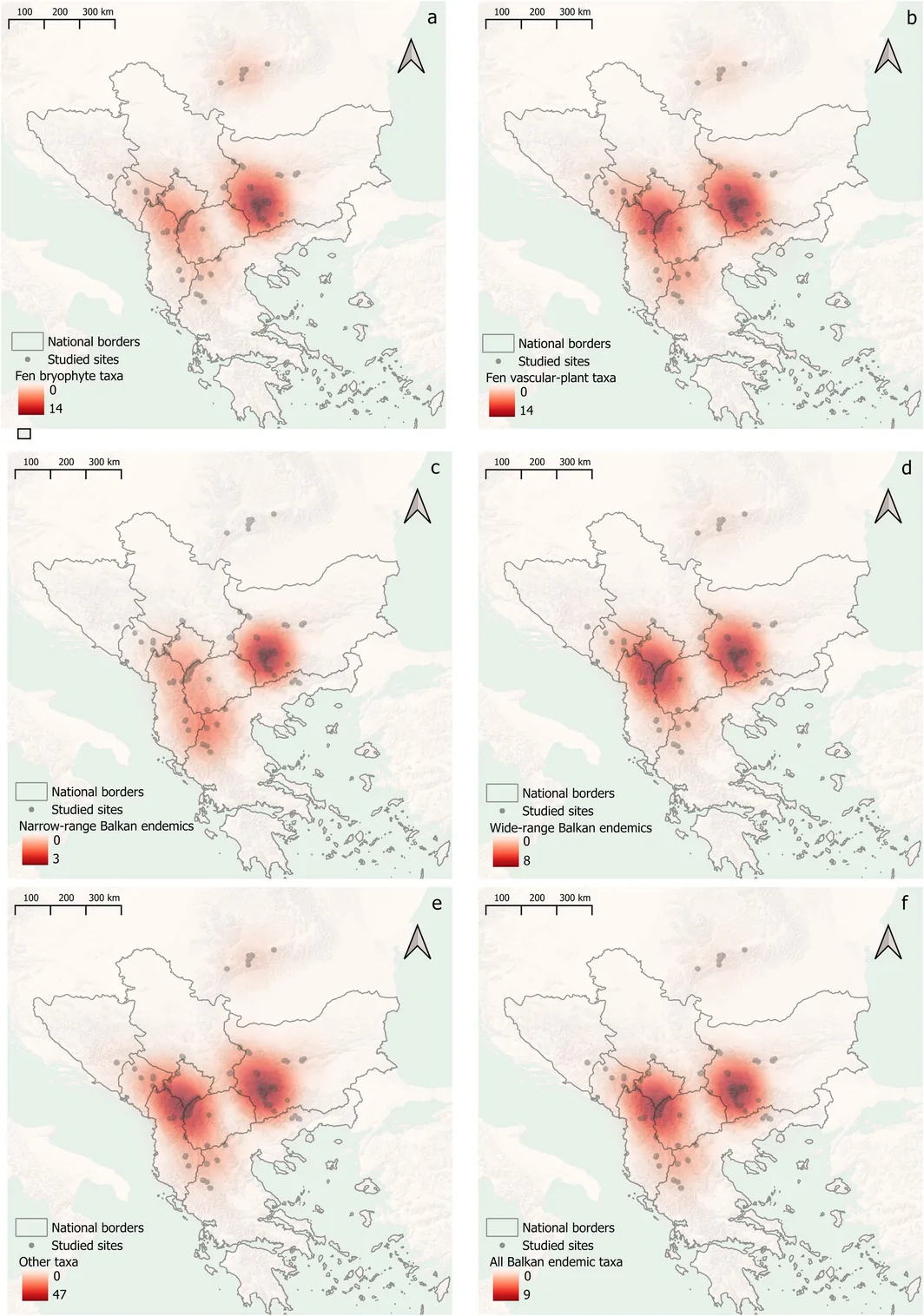
Hotspots of fen plant endemism in the Balkans, mapped using kernel density estimation of point‐based relevé data with a uniform 50 km bandwidth: (a) fen bryophyte taxa, (b) fen vascular‐plant taxa, (c) narrow‐range Balkan endemics, (d) wide‐range Balkan endemics, (e) other taxa and (f) all Balkan endemic taxa. Small circles indicate study sites. Zero density refers to areas with no taxa from the groups recorded, either due to their absence in the plots or a lack of data. As only relevés with at least one endemic taxon present were included, resulting in an altitudinal restriction to areas above approximately 1,000 m a.s.l., the maps show the complete pattern for endemic groups (c, d, f), but not for other groups whose richness at lower elevations is not mapped.

### Pattern across the past and present environment

The palaeoclimate data indicate that, at sites with Balkan fen endemics, past climate extremes mainly occurred during the Late Pleniglacial and the Early Holocene (Fig. 3). The lowest summer temperatures typically coincided with the LGM (median negative deviation from the recent value of 5.3 °C), whereas the lowest winter temperatures (−7.3 °C) and annual precipitation (−11%) were most often modelled for the Late Pleniglacial. Peak winter and annual precipitation occurred during the Bølling–Allerød interstadials, with increases of up to 63% and 42%, respectively. The highest summer temperatures (+2.5 °C) were consistently modelled for the Early Holocene (ca. 9050 BP). Minimum precipitation was most frequently modelled for the periods between Early Holocene (summer) and Late Pleniglacial (winter), with reductions of up to 70% in summer and 15% in winter. Maximum summer precipitation (up to +85%) varied spatially and most often occurred during the Common Era (ca. 750–1550 BP). Winter temperatures are currently at their highest.

**Fig. 3:**
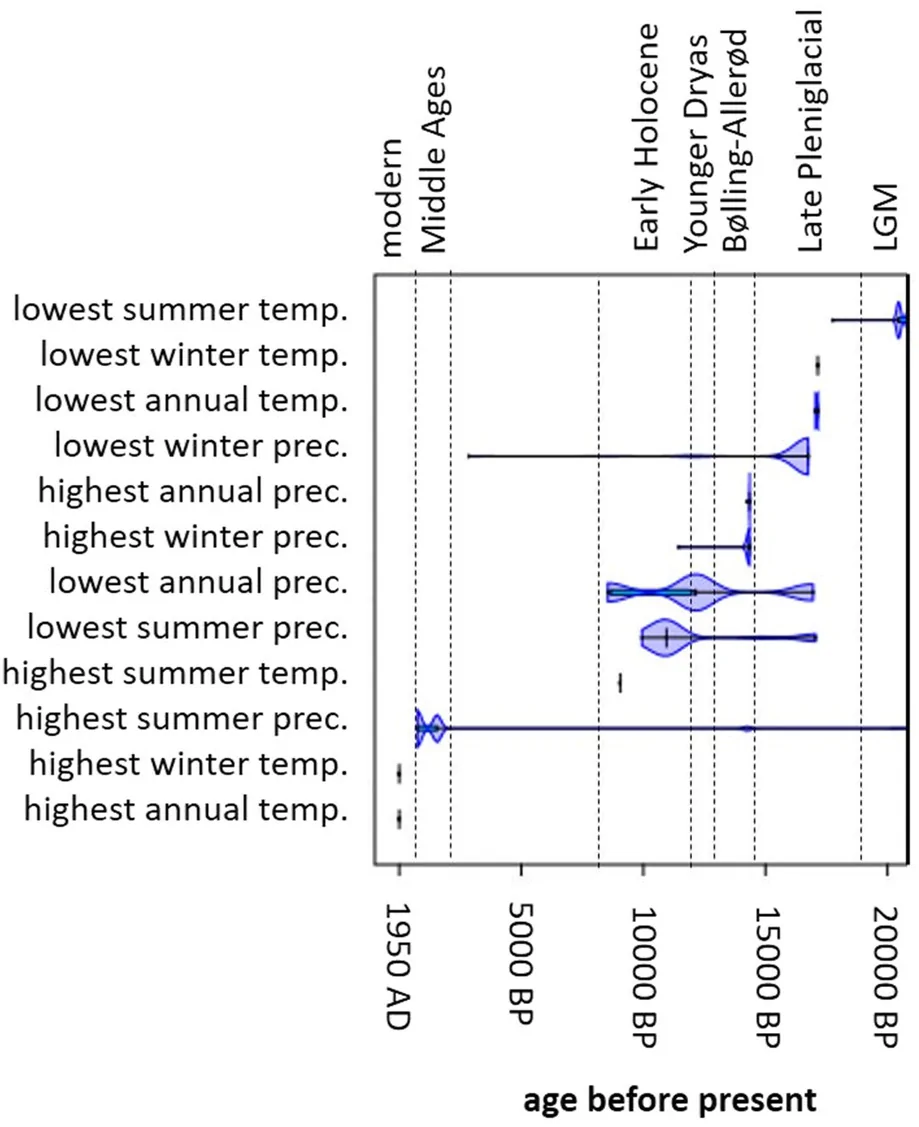
Violin plot with notches showing when the maximum or minimum palaeoclimatic deviations occurred in the last 20,000 years.

According to Spearman's rank correlation (Table 1), the richness of both wide‐range and narrow‐range Balkan endemics showed significant positive correlations with some elevation‐related variables and with the recent sum of winter precipitation; these results were also maintained in SAR_err_ (Fig. 4). There was no correlation with adjusted pH. Richness of wide‐range and all endemics further correlated negatively with water level (EIV), again kept in SAR_err_. Palaeoclimatic variables showed strong correlations with current richness of narrow‐range endemics, but not with that of wide‐range endemics. The positive correlation between the magnitude of past annual precipitation increase (peaking in Bølling–Allerød interstadials) and the richness of narrow‐range endemics was the strongest in the entire dataset and persisted in SAR_err_ as well. Richness of narrow‐range endemics was also positively correlated with other variables, such as overall climate stability, but these relationships were not significant in SAE_err_. When focusing on non‐endemic fen specialists, different factors correlated with the richness of specialised bryophytes and vascular plants. The richness of fen vascular plants was significantly higher at flatter relief, in moister conditions and under warmer climates; SAR_err_ confirmed these results. On the contrary, the richness of bryophytes correlated with a wide set of edaphic, climatic and palaeoclimatic factors. The strongest correlation was found for the past winter precipitation increase and was negative. No climate or palaeoclimate variable remained significant for bryophyte richness in SAR_err_, in which only statistically significant negative correlation with adjusted pH and positive correlation with minimum elevation within a 10 km radius were retained. The richness of other (matrix‐derived) taxa correlated most strongly with edaphic factors, positively with adjusted pH and negatively with water level (EIV) and elevation.

**Table 1:** Spearman's correlation coefficients between individual explanatory variables and the richness of individual plant groups.

	fen specialists		Balkan fen endemics			other
	bryophytes	vascular plants	narrow range	wide range	all	
**Environmental factors (*P* values adjusted)**						
*Recent climate*						
Ann temp (recent)	−0.16*	0.26***	−0.2**	−0.24***	−0.3***	n.s.
Summ temp (recent)	n.s.	**0.29** ***!	−0.19**	−0.28***	−0.34***	n.s.
Wint temp (recent)	−0.27***	0.19**	n.s.	−0.18*	−0.21**	n.s.
Annual prec (recent)	−0.19**	n.s.	n.s.	n.s.	n.s.	n.s.
Summ prec (recent)	0.16*	n.s.	−0.32***	n.s.	n.s.	n.s.
Wint prec (recent)	−0.28***	n.s.	**0.19** **!	**0.19** **!	**0.26** ***!	n.s.
*Palaeoclimate dynamics*						
Ann temp (past decrease)	n.s.	n.s.	−0.51***	n.s.	−0.22***	0.17*
summ temp (past increase)	0.21***	n.s.	n.s.	n.s.	n.s.	n.s.
Summ temp (past decrease)	−0.21**	n.s.	−0.34***	n.s.	n.s.	n.s.
Wint temp (past decrease)	0.26***	n.s.	−0.38***	n.s.	−0.26***	n.s.
Annual prec (past increase)	n.s.	n.s.	**0.56** ***!	n.s.	0.18**	n.s.
Annual prec (past decrease)	0.3***	n.s.	−0.27***	n.s.	−0.23***	n.s.
Summ prec (past increase)	0.23***	n.s.	0.23***	n.s.	n.s.	n.s.
Summ prec (past decrease)	n.s.	n.s.	0.53***	n.s.	0.21***	n.s.
Wint prec (past increase)	−0.37***	n.s.	n.s.	n.s.	n.s.	n.s.
Wint prec (past decrease)	0.33***	n.s.	0.18*	n.s.	n.s.	n.s.
Overal climate stability	0.22***	n.s.	0.29***	n.s.	n.s.	n.s.
*Edaphic factors*						
Water level (EIV)	n.s.	**0.17** *!	n.s.	**−0.23** ***!	**−0.16** *!	**−0.56** ***!
Adjusted pH	**−0.33** ***!	n.s.	n.s.	n.s.	n.s.	**0.34** ***!
*Topographic factors*						
Elevation	n.s.	−0.28***	**0.38** ***!	0.29***	0.42***	**−0.21** **!
Mean TRI (5 km)	n.s.	**−0.36** ***!	0.22***	0.26***	0.32***	n.s.
Minimum elevation (10 km)	**0.2** **!	n.s.	n.s.	0.17*	0.21***	n.s.
Elevation >1700 m (5 km)	n.s.	n.s.	0.3***	**0.36** ***!	**0.45** ***!	**n.s**.
**Plot size (unadjusted *P* values)**						
Relevé plot size	n.s.	0.14**	−0.2***	n.s.	**−0.16** **!	0.19***

Bold values with the ‘!’ symbol indicate which of these relationships remained the same and remained statistically significant when controlling for multicollinearity and spatial autocorrelation (SAR_err_ models). ann = annual, summ = summer, wint = winter, temp = temperature, prec = precipitation, past decrease/increase = the magnitude of the difference between the recent and the past minimum/maximum value, (× km) = the diameter of the radius in which the value was calculated, elevation > the area of elevation above the threshold in the radius.

* *p* < 0.05.

** *p* < 0.01.

*** *p* < 0.001.

**Fig. 4:**
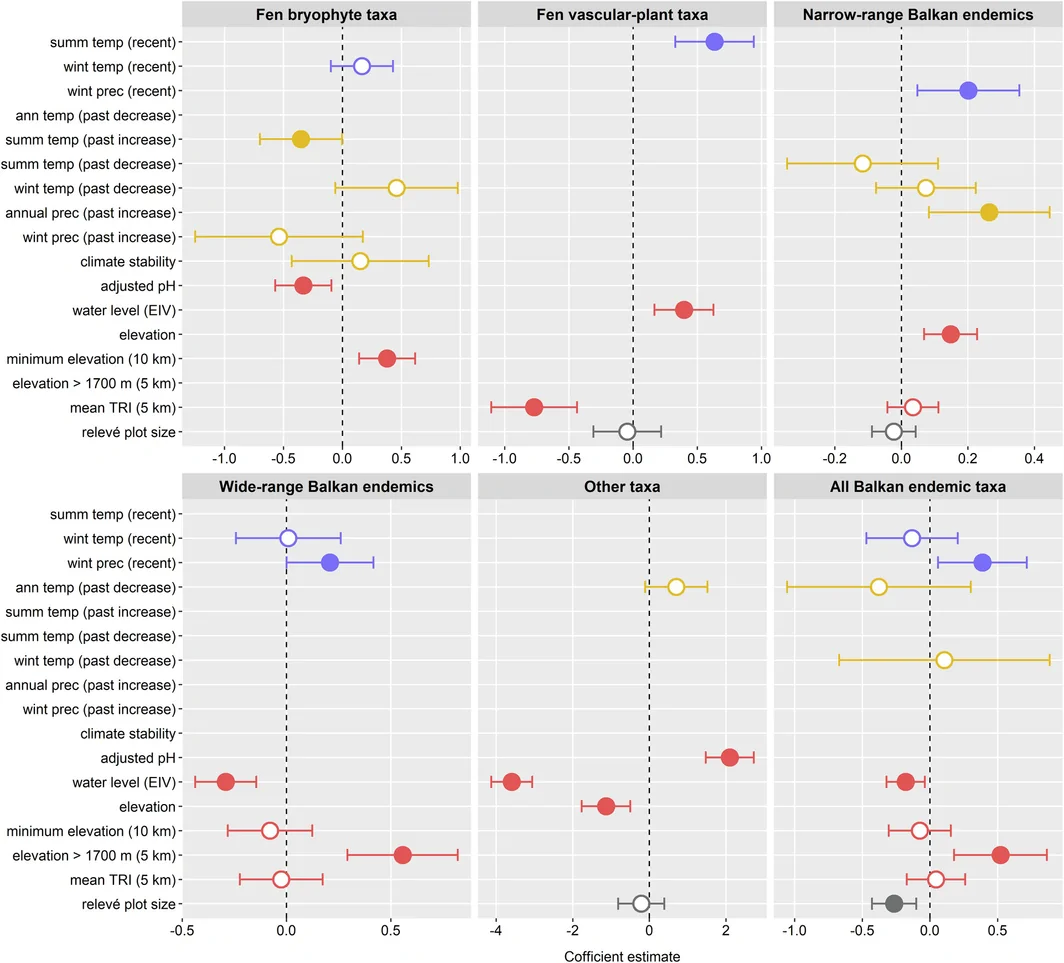
Results of Spatial Autoregressive Error Models for selected predictors of recent taxon richness. Dots show coefficient estimates with standard errors; filled dots indicate significant effects (*P* < 0.05). Missing symbols denote variables excluded due to insignificant or collinear relationships. Colours indicate predictor groups: current climate (blue), past climate deviations (yellow), soil and topography (red) and plot size (grey).

Plot size was significantly positively correlated only with the richness of non‐endemic fen vascular plants and matrix‐derived taxa, even without adjustment for multiple testing. In contrast, it was negatively correlated with the richness of Balkan endemics, and this relationship was retained in the SAR_err_ models.

The first PCA axis of abiotic factors (Fig. 5) extends from the highest eastern mountains to other areas and largely coincides with palaeoclimate variables and pH. The two‐axis space can be interpreted well in a diagonal view, with one diagonal stretching from the east (bottom left) to the southwest (upper right); the northwest is less climatically stable, showing a larger difference between current and past minimum temperatures (LGM) and currently experiencing higher precipitation. The second diagonal follows the elevational gradients from the warm, base‐rich low‐elevation areas across the Balkans to the high, acidic Southern Carpathians. The palaeoclimate is crucial to this gradient, because when it is not included in the analysis, the Southern Carpathians are no longer delimited from the Balkans (Fig. S4). Colder winters during glacials and a drier climate during the Late Pleniglacial and the Early Holocene characterise the South Carpathians against the Balkans. The richness of Balkan endemics peaks in the middle part of the second axis, where the Rila and Korab hotspots are located. See Fig. S5 for the distribution of individual taxa along the abiotic PCA gradients, Fig. S6 for the pattern across regions and Appendix S2 for the detailed interpretations.

**Fig. 5:**
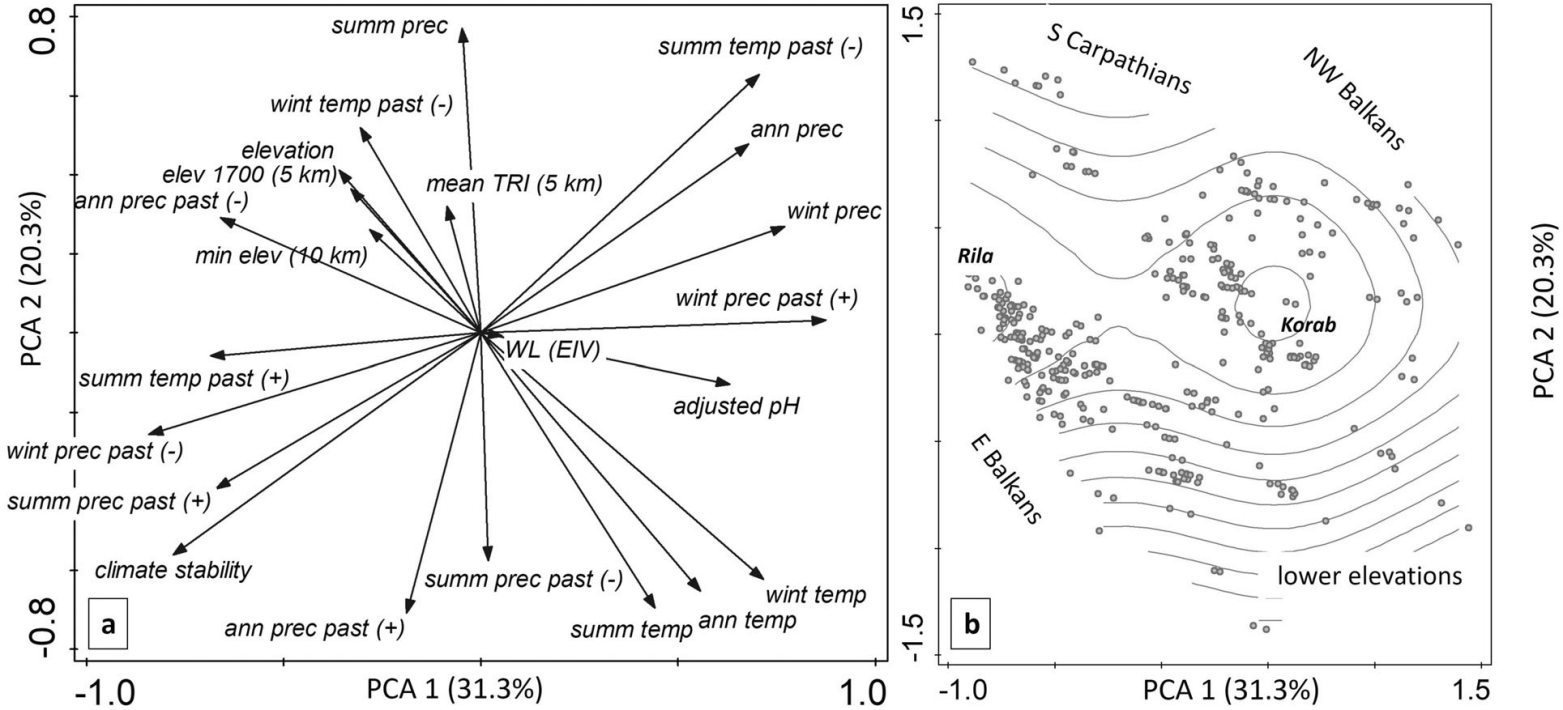
The first two axes of PCA: (a) scatter plot of the abiotic variables, (b) scatter plot of sites with isolines of richness of Balkan fen endemics (GAM). *ann* = annual; *minus* = absolute value of maximum negative deviation; *past =* past deviation from the recent value; *plus* = maximum positive deviation; *prec* = precipitation sum; *summ* = summer; *temp* = mean temperature; *wint* = winter.

When species composition of Balkan endemics was seen as focal and environmental variables as explanatory ones (CCA; Fig. 6), the first axis showed again the east‐to‐west gradient correlating with palaeoclimate, mountain massiveness and pH, while the second axis differentiated more western locations in the central Balkan area (*Pedicularis olympica* Boiss., *Crocus pelistericus* Pulević, *Epilobium vernonicum, Silene asterias*) and the more southern serpentinite areas with higher pH (*Carex castroviejoi, Pinguicula hirtiflora, Narthecium scardicum*).

**Fig. 6:**
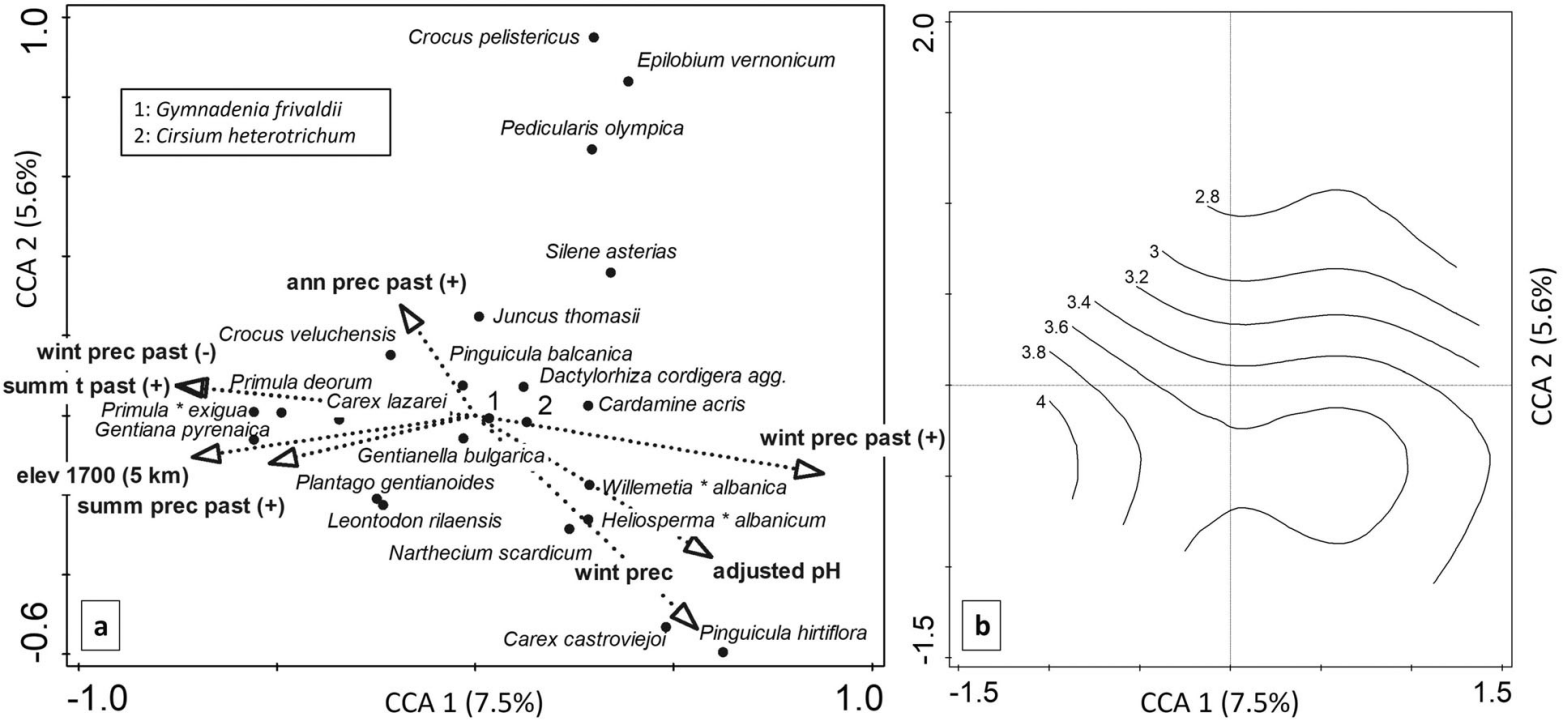
The biplot of CCA testing the effects of explanatory abiotic variables on the occurrence of endemic taxa in the plot (a), with GAM isolines of plot‐scale endemic richness plotted *a posteriori* onto the CCA scatter (b). See caption of Fig. 5 for explanation of abbreviations.

All presented GAMs showing patterns in endemic richness along the PCA and CCA axes were statistically significant (*p* < 0.001).

## DISCUSSION

### The community‐scale perspective on Balkan fen endemics

Our quantitative, community‐level, pan‐Balkan synthesis addresses the neglected phenomenon of fen plant endemism in Europe. Contrary to the preliminary checklist of European wetland plant endemics (Hobohm & Bruchmann 2011), we listed 17 additional previously omitted taxa (see Table S1, *e.g. Narthecium scardicum*, *Gymnadenia frivaldii* Hampe ex Griseb., *Cirsium heterotrichum*) and tested the determinants of endemism patterns at the community level. We argue that co‐existence within local communities indicates long‐term regional occupancy, prolonged adaptation to inter‐specific interactions and local edaphic conditions (Hájková *et al*. 2008), as well as sufficient time for colonisation of individual fen and spring islands. These processes result in up to nine co‐existing endemic taxa. Follow‐up research could identify the borders between the standalone Balkan and widespread vegetation and habitat units (Peterka *et al*. 2017; Chytrý *et al*. 2020). Within the Balkan vegetation units, the reported east–west gradient associated with palaeoclimate matches the phytosociological view of de Foucalt (2022), who considers the east‐Balkan high‐mountain fens, together with the South Carpathian and Anatolian fens, to form a separate vegetation alliance (*Pinguiculo‐Plantaginion gentianoidis*). Ecologically, eastern fens more often occupy silicate bedrock, resulting in acidic, less mineral‐rich conditions than south‐western fens. However, species diagnostic of south‐western fens, such as *Narthecium scardicum*, may also occur in strongly acidic and non‐calcareous conditions characterised by acidophile peat mosses, especially in the Prokletije Mts (Treskavitsa reka in Montenegro and Sylbica‐Doberdol area in Albania) and the Jablanitsa Mts (Podgorecko lake in North Macedonia). Generally, pH and bivalent bases (here combined as adjusted pH; Plesková *et al*. 2016) accounted for the most significant overall compositional differences, as observed in other studies worldwide (Malmer 1986; Vitt & Chee 1990), but only poorly explained the distribution of Balkan endemics and their concentration at the community scale (see also Hájková *et al*. 2006). The exceptions are *Pinguicula hirtiflora*, *Carex castroviejoi, Cirsium epiroticum* and *Heliosperma pusillum* subsp. *albanicum* (K. Malý) Niketić & Stevan., all of which prefer alkaline springs and spring fens.

### Community richness of Balkan fen endemics

If the role of pH is not as significant for Balkan endemics as for other plant groups, which factors then explain the community‐level diversity pattern for Balkan fen endemics and other specialised taxa? One could expect that current climate conditions control competitive interactions within a community (Hájek *et al*. 2022; Lyu & Alexander 2022), thereby influencing community‐scale co‐existence. Indeed, we show that the richness of Balkan endemics increases with winter precipitation (the pattern persists even after controlling for spatial autocorrelation) and decreases with temperature. Winter precipitation, which gradually infiltrates into groundwater after snowmelt, ensures sufficient groundwater in aquifers throughout the summer. Moreover, it supports snow beds, which may serve as secondary habitats for some Balkan fen endemics such as *Crocus veluchensis, Plantago gentianoides* Sibth. & Sm. and *Scorzoneroides rilaensis* (Hayek) Holub (Hájková *et al*. 2006), thereby supporting regional metapopulations. Summer precipitation, whose total correlated negatively with narrow‐range endemics, is largely lost to runoff during heavy summer storms and may even contribute to the erosion of fen peat. Palaeoclimate variables were the strongest determinants, but only for narrow‐range endemics. Such endemics are concentrated more where Bølling–Allerød annual precipitation extremes were higher and where either annual cold extremes were milder during the glacial or summers were cooler. Summer temperature is a key factor distinguishing tundra from steppe and temperate biomes (Archibold 2012). Given this, our results support the hypothesis that during the LGM, the narrow‐range Balkan endemics persisted under cooler tundra conditions rather than the warmer steppe‐tundra or mountain‐steppe conditions that prevailed in southeastern Europe (Tonkov 2021). In the southern part of the western hotspot area (Fig. 2c), characterised by ultramafic substrates, glaciers were present during the LGM (Leontaritis *et al*. 2022), similar to areas further north. The stable tundra climate likely supported *N. scardicum* and the strictly serpentinite‐associated *C. castroviejoi*. The latter might have potentially utilised ultramafic substrates in Lake Ohrid's temperate refugia (Sadori *et al*. 2016), which is supported by our Jablanitsa Mts record. For *C. castroviejoi* and warmth‐demanding endemics (*P. hirtiflora, C. epiroticum*), milder cold extremes and higher precipitation were likely more critical for survival than summer temperatures.

The significance of palaeotemperature variables disappeared in SAR_err_, suggesting that such tundra‐like conditions were restricted to a few distinct, larger regions, corresponding to the recent diversity hotspots, rather than small islands scattered across the Balkans. For oceanic islands worldwide, Weigelt *et al*. (2016) indeed demonstrated that island size during the LGM played a key role in shaping their current richness of endemic taxa, whereas the effect of changes in isolation since the LGM was not significant. Nevertheless, Hammoud *et al*. (2021) demonstrated that bridging the island to the mainland decreased endemism, illustrating that persistent isolation is important. Later, the narrow‐ranged endemics probably increased metapopulation sizes in areas where the Bølling–Allerød interstadials were wetter, thereby increasing the chance of their survival during the dry Younger Dryas and Early Holocene (Wright Jr *et al*. 2003; Kuhlemann *et al*. 2009). Northwards, where narrow‐range Balkan endemics are missing (southern Carpathians, SE Dinarids), the climate was less stable and experienced much drier summers in the early Holocene than recently (but not as high as Bølling–Allerød peak) and lower winter temperatures in the LGM (see Fig. 5a).

The areas rich in Balkan endemics probably always acted as islands in otherwise drier or warmer, and in interstadials even forested, landscapes (Tonkov 2021). The continual existence of streams, springs and fens, closely linked to past precipitation patterns that proved extraordinarily important in our study, was obviously a prerequisite. Climate stability is generally a key condition for endemism, as demonstrated by large‐scale studies (Jansson 2003; Harrison & Noss 2017; Cai *et al*. 2023). In our study, its effect is relatively small because of the positive effects of past precipitation anomalies (*i.e*. instability). The magnitude of cold and dry climate extremes, however, correlated negatively with the richness of narrow‐range endemics in most cases. The high‐mountain islands of wet tundra, hence, might develop their own endemic taxa, such as *Primula deorum* in the Rila Mts, the ancestral taxon of the *Auricula* section of probable Quaternary origin, with the most closely allied non‐wetland taxon *P. glutinosa* Jacq. occurring in the Alps and rarely also the Dinarides (Boucher *et al*. 2016). More commonly, however, nearby mountain islands share the same endemic taxon nowadays: *Primula frondosa* subsp. *exigua* (allied with *P. farinosa* L., *P. scotica* Hook. and *P. halleri* J. F. Gmel.; Guggisberg *et al*. 2006) is shared among neighbouring Rila, Pirin, and Vitosha Mts (Bulgaria), *Narthecium scardicum* among the south‐western mountain ranges, and *Pedicularis olympica* and *Epilobium vernonicum* among the neighbouring South‐Central mountain ranges (Kaimakchalan, Pelister, Jablanitsa). The pattern of nearby islands sharing the same endemics could arise from long‐distance dispersal after the endemic taxon had evolved in a single island; such a scenario is expected not only for oceanic archipelagos such as Macaronesia (Fernández‐Palacios *et al*. 2024), but also for endemic dryland taxa of the Balkan mountain system like *Campanula orbelica* Pančić (Ronikier *et al*. 2023).

For more widespread Balkan endemics, however, individual high mountains might not have been islands during the glacials; instead, they might have experienced a single continuous distribution range in the Balkans and eventually Anatolia, isolated from more northerly and westerly areas by the glaciers (the Alps, southern Carpathians) or by forest‐steppe (Transylvania, Pannonia). During the warm and dry period of the Early Holocene, they might also persist at lower elevations, as indicated by their drought tolerances (reflected in the ecological indicator values for water level, Table 1, Fig. 4). In interglacials, including the Holocene, they spread to higher elevations, where they meet narrow‐range endemics, forming the extreme community‐scale richness of Balkan endemics, as in the Rila and Korab Mts (Table S4). As a result, their local richness does not correlate with palaeoclimate, mirroring the non‐endemic fen specialists. The recent climate parameters instead shape their richness, but elevation *per se* shows the strongest correlations persisting in SAR_err_. The stronger competitive relationships at lower elevations, which experience a longer vegetation period and higher nutrient availability (Sommer *et al*. 2010; Hájek *et al*. 2022), might explain this result. Additionally, grazing intensity is moderate in high mountains, further supporting local co‐existence. Moreover, lower elevations harbour edaphically extreme habitats (such as petrifying spring fens or poor fens) which often lack Balkan endemics (Hájek *et al*. 2008).

### Richness patterns for non‐endemic specialised fen taxa

Fen bryophyte richness paralleled the pattern of narrow‐range endemics, with the significant effects of palaeoclimate variables and the positive effects of overall climate stability and summer‐cold and winter‐dry conditions during the pleniglacial. These results suggest they probably utilised the same tundra‐like refugia. This pattern, however, did not lead to endemism, as bryophytes generally show much lower spatial genetic structure than angiosperms due to the long‐distance dispersal by spores (Patiño & Vanderpoorten 2018). A key difference from narrow‐range endemics is that bryophyte richness shows a negative relationship with winter precipitation, both recent and past. This pattern disappeared in SAR_err_, suggesting possible covariance with another unmeasured coarse‐scale factor. Nevertheless, the eventual ecological explanations may involve regional extinctions caused by altered snow conditions affecting bryophyte taxa differently—either through reduced light availability under increased snow cover (Yu *et al*. 2024), or through increased frost damage under reduced snow cover (Küttim *et al*. 2019).

By contrast to bryophytes, the richness of non‐endemic fen vascular plants correlated with few explanatory variables—negatively with elevation, while positively with temperature and water level. This difference between bryophytes and vascular plants supports the previous pan‐European study by Jiménez‐Alfaro *et al*. (2023), who reported that macroclimate has a greater effect on shaping richness patterns for bryophytes than for vascular plants. On the other hand, the effect of pH on vascular plants (both non‐endemic and endemic) was lower in our study than expected from the study by Jiménez‐Alfaro *et al*. (2023). The reason may be that the pH niches of fen plants are generally broader in the Balkans than in some other European regions (Hájková *et al*. 2008), and in arctic–alpine climates than in warmer ones (Hájek *et al*. 2022). The pattern for other, that is, matrix‐derived, taxa is not surprising and reflects the well‐known trend of a higher number of non‐fen taxa in drier, that is less extreme, fens (Horsáková *et al*. 2018) and in more alkaline conditions, where the surrounding calcareous areas support a larger species pool (Pärtel 2002).

### A continuum between narrow‐ranging and wide‐ranging Balkan fen endemics

Despite broad distribution ranges across the Balkans, the taxa classified as wide‐range endemics in our study are still clearly associated with alpine and subalpine zones. They might have evolved within a single mountain range but spread across a wider area, as confirmed in the above‐mentioned case of the dryland taxon *Campanula orbelica* (Ronikier *et al*. 2023). This scenario may also apply to *Scorzoneroides rilaensis*, which is common in the Rila Mts but shows isolated populations in the West Balkans (Dimitrov in von Raab‐Straube & Raus 2021; this study) and in the southern Carpathians (Coldea *et al*. 2009). Another taxon with pappus‐bearing seeds, *Cirsium heterotrichum*, shows a clustered distribution along the principal environmental gradients but a disjunct distribution. It occurs frequently in the Vitosha and Western Stara Planina Mts, but not in the neighbouring Rila Mts, which shares with the Vitosha Mts some other fen‐endemic taxa and disjunctly occurring coldland taxa such as *Trichophorum cespitosum* (L.) Hartm. Then it occurs in the analogous vegetation in the distant area of the Prokletije Mts, where it prefers the acidicole *Sphagnum*‐fens resembling the Bulgarian ones (own unpublished data) and in the areas of Šar and Korab Mts. The other areas of distribution represent the serpentinite ecotype occurring in the Valamara Mts, and acidicole yet morphologically distinct populations in the Jablanitsa and Pelister Mts, sometimes considered as separate taxon *Cirsium halacsyi* Formanek. This distribution pattern may suggest environmental filtering and multiple long‐distance colonisation events which, however, are not frequent enough to prevent phenotypic and ecotypic local adaptations. A similar pattern occurs for *Pedicularis olympica*, which we classified as a narrow‐range taxon due to its geographically clustered distribution in our study area and the taxonomic uncertainty of some disjunct populations (see Table S1). Another taxonomically unclear case is *Gentiana pyrenaica* L., which shows a disjunct distribution with restricted occurrences in the East Balkans, northeast Anatolia and the Caucasus and distant populations in the Pyrenees (the taxonomic opinion adopted in Euro+Med PlantBase, Euro+Med 2006), unless the Bulgarian and further eastern populations are assigned to *G. dhsimilensis* K. Koch (taxonomy following POWO 2026).

Not all Balkan narrow‐ or wide‐range endemics might have evolved in the Balkans. The alternative scenario for how a taxon became a Balkan endemic posits that a formerly widespread taxon became extinct in most of its range, leaving relict populations in the Balkans. This palaeoendemic scenario, not uncommon for endemics (Cai *et al*. 2023), is expected for non‐fen Balkan endemic species of damp rocks, such as *Haberlea rhodopensis* Friv. (Petrova *et al*. 2015), and may also apply to *Primula deorum* as an ancestral taxon within the section *Auricula* Duby (Boucher *et al*. 2016). The formerly widespread range, restricted during the last glacial period, was even confirmed palynologically for the heathland Balkan endemic *Bruckenthalia spiculifolia* (Salisb.) Rchb., which extends into mires (Hájková *et al*. 2006). Once widespread across Europe during interglacials and even during early Weichselian, *B. spiculifolia* became extinct during the Last Pleniglacial in most of its former range (Birks 2019). The association with moist, cold, but not frosty areas is further implied by the pattern that most of its fossil pollen records come from northwest Europe (Birks 2019). In the context of Balkan fen endemism, the story of *Bruckenthalia* suggests a possible explanation for the origin of *Narthecium scardicum*, an emblematic endemic of the Southwest Balkans. Together with the Corsican *N. reverchonii* Čelak. and the Caucasian *N. balansae* Briq., which also occurs in Anatolia (Ağaçbaşı Yayla), it represents a group of small‐range vicariants of the morphologically and ecologically similar *N. ossifragum*, characterising NW European oceanic mires with historical isolated occurrences in more continental Central European areas. A glacial fragmentation of the ancestor's wide range cannot be ruled out. The distinction between the neoendemics evolving in a single Balkan mountain island, that is, initially narrow‐ranged, and the once widespread palaeoendemics may query the appropriateness of designating the fens rich in Balkan endemics as ‘Relict mires’, as in the EUNIS habitat classification.

### Non‐intuitive adverse effects of plot size

The lack of significant positive effects of plot size on the community richness of individual groups of taxa (SAR_err_), and the significant negative correlations with plot size for Balkan endemics and fen specialists, are surprising and counterintuitive results. The explanation probably includes natural and sampling effects. Small plot sizes were used whenever the fen or spring patch was too small to accommodate the standard plot size; in some locations, there was no other patch to sample. It seems that the small patch size did not prevent the Balkan endemics and fen bryophytes from co‐existing with the local species pool, but did limit the accidental occurrence of matrix‐derived taxa entering the fen or spring from the surroundings. In contrast, larger plot sizes were sampled at lower elevations (database data), where the species pool of Balkan endemics is smaller but the fen area is usually larger. These patterns had resulted in the observed negative relationship between plot size and richness of Balkan endemics and fen bryophytes. The absence of the expected species–area relationships within our plot size range has positive implications. First, variable plot size does not substantially confound the reported patterns. Second, even small patches usually harbour a substantial proportion of the local pool of endemic taxa, suggesting a low level of competitive exclusion. However, whether this fine‐scale co‐existence helps populations survive environmental changes is unclear, as extinction risks are generally higher in small populations.

### Conservation perspective: Insularity, ongoing climate change and landscape management

Our results suggest that island biogeography principles help explain the main observed patterns. The comparison between narrow‐ and wide‐range endemics, however, indicates a complex hierarchical structure of insularity. The entire area of the Balkan mountain ranges may be viewed as one large island, with individual highest mountains as islands within the island (Cordeiro *et al*. 2025), and individual fen sites as small islets, which are also undergoing rapid decline and fragmentation. Results from calcareous fens in the Western Carpathians demonstrated that individual small fens may still act as habitat islands, whose local richness is influenced by their age (Horsák *et al*. 2012) or proximity to large and species‐rich patches (Mendez‐Castro *et al*. 2021), which support species capable of long‐term persistence rather than dispersal ability (Conti *et al*. 2022) and may even support genetic diversification among them (Mikulášková *et al*. 2026). Nevertheless, this lowest hierarchical level is vulnerable to rapid climate change, which may lead to the disappearance of many small springs and fens, many of which are already affected by spring tapping and drainage. These local extinctions may soon be reflected at higher levels, and fen endemism in the Balkans may be on a trajectory towards extinction.

Although many high‐mountain fens, but not all, are part of National Parks according to IUCN categorisation or at least Natura 2000 sites, their future is not guaranteed by a non‐intervention regime. Apart from preventing water withdrawal, finding the optimal grazing mode and intensity is therefore a key challenge. MH, PH and IA recorded the exceptional richness of Balkan endemics per relevé (up to eight taxa; Table S4) in the fens of the Rila Mts, which were subjected to moderate grazing by semi‐wild horses. Continued moderate grazing disturbance may help stabilise mountain fens by supporting local co‐existence and preventing succession, which could be accelerated by climate change (Hartmann *et al*. 2025). In contrast, overgrazing by large herds of domestic animals may have a detrimental effect, especially in small fens, by causing excessive disturbance and eutrophication (Bergmeier 2023).

Ongoing climate change, particularly the rapid rise in summer temperatures and increased drought, is raising concerns about the future of Balkan wetlands (Malatesta *et al*. 2023; Shumka *et al*. 2024). However, our results suggest that Balkan fens experienced even warmer and simultaneously drier summers than today during the last 21,000 years. Currently, the Balkan Mountains experience an increase of approximately 2 °C in summer temperatures compared with the baseline period 1961–1990 (Burić 2024; data from the Žabljak meteorological station, 1450 m), approaching the Early Holocene historical maxima, which are likely to be surpassed within the next decades (Burić 2024). However, our results suggest that precipitation dynamics since the LGM may have a greater influence on recent patterns of endemic richness. At present, summer and annual precipitation totals do not decline to the low levels reconstructed for the Early Holocene, and summer precipitation may even increase in some Balkan high mountains (*e.g*. at the Lazaropole station in North Macedonia; Radevski *et al*. 2024). Conversely, summer precipitation is more likely to occur as torrential rain (Valcheva & Spiridonov 2023), causing fen erosion and high run‐off rather than groundwater infiltration, which is further hindered by high evapotranspiration. Our results indicate that a warmer, though not significantly drier, climate (a prediction that disregards increasing runoff and evapotranspiration) might hamper specialised fen bryophytes more than wide‐ranging Balkan endemics. This highlights the close association of fen bryophytes with specific refugia and their great vulnerability to ongoing warming (Rehell & Virtanen 2016; Horsáková *et al*. 2018; Küttim *et al*. 2019). Sopotlieva *et al*. (2024) revisited several early‐sampled fen sites in the Vitosha Mts and indeed reported changes in the bryophyte layer, but not in the representation of Balkan endemics. Even amid rapid climate change, we may not lose Balkan fen endemics if we avoid human interventions in the natural water regime. Afforestation of groundwater recharge areas, which may increase transpiration and thus prevent infiltration of rainwater into the aquifer during dry periods, as well as direct groundwater extraction for drinking water, snowmaking or replenishment of mountain lakes and ponds, should be strictly avoided in the most important fen areas. Fen endemics should be included in international schemes for the legal protection of species (*e.g*. the European Union's Habitats Directive). Their habitats and communities should be monitored for both successional changes and connectivity, and a transboundary approach should be adopted for this monitoring and conservation planning. Avoiding direct destruction and hydrological interventions in their catchments, together with the restoration of drained, afforested, eroded or overgrazed areas, will greatly increase the chances that these unique habitats will survive ongoing climate change.

## AUTHOR CONTRIBUTIONS

MH and PH conceived the idea for the paper. Together with IA, they initiated the earliest research in Bulgaria and ultimately collected the largest number of relevés across the study area. All other authors, except JD, DSt and PB, led or contributed to field research in different parts of the study area (for regions, leadership and numbers of sites, see Table S2). PH identified bryophytes from most vegetation plots. JD extracted palaeoclimatic data and calculated SARerrs. MCh prepared all maps. TP and RČ managed the literature and L. Melovski's data, respectively. PB identified critical specimens of Cirsium and provided original taxonomic insights into this genus. LS carried out the same work for the genera Crocus and Pinguicula and prepared photographic panels for the Supporting Information. DSt provided regional expertise for Montenegro. MH led data analyses and writing. ZP led editing and formatting. All co‐authors contributed to the writing and editing, or critically reviewed earlier versions of the paper.

## FUNDING INFORMATION

MH, PH, ZP and TP were supported by the Czech Science Foundation (project no. GA24‐10844S). PH was further supported by the long‐term developmental project of the Czech Academy of Sciences (RVO 67985939).

## Supporting information


**Figure S1.** Photographs of endemic taxa characterising Balkan high‐mountain fens: (a) *Narthecium scardicum*, (b) *Pedicularis olympica*, (c) *Primula deorum*, (d) *Primula frondosa* subsp. *exigua*, (e) *Pinguicula hirtiflora*, (f) *Gentiana pyrenaica*, (g) *Plantago gentianoides*, (h) *Gymnadenia frivaldii*, (i) *Heliosperma pusillum* subsp. *albanicum*, (j) *Dactylorhiza cordigera*, (k) *Willemetia stipitata* subsp. *albanica*, (l) *Carex lazarei*, (m) *Silene asterias*, (n) *Cirsium heterotrichum*, (o) *Crocus pelistericus*, (p) *Gentianella bulgarica*, (q) *Juncus thomasii* and (r) *Pinguicula balcanica*.

**Figure S2.** The results of the Twinspan classification, diagnostic taxa of individual clusters and the association of individual endemic taxa with the vegetation types.

**Figure S3.** Illustrative photos of spring and fen vegetation with Balkan endemics: (a) *Primuletum exiguo‐deori*, (b) *Willemetietum stipitatae*, (c) *Dactylorhizo cordigerae‐Eriophoretum latifolii*, (d) *Cratoneurion commutati*, (e) *Pinguicula hirtiflorae‐Cratoneuretum* and (f) *Narthecion scardici*.

**Figure S4.** Ordination diagram of PCA without palaeoclimatic variables.

**Figure S5.** Individual endemic taxa plotted in PCA of environmental variables.

**Figure S6.** PCoA of the region‐based frequency table of taxa.

**Table S1.** List of Balkan endemic and subendemic vascular plant taxa narrowly associated with fen and spring habitats.

**Table S2.** Data sources.

**Table S3.** Synoptic table of vegetation types.

**Table S4.** Community richness statistics of endemics for 11 regions.

**Appendix S1.** Extended Materials and Methods.

**Appendix S2.** Complete results of ordination analyses.

## Data Availability

Selected data and script that support the findings of this study are openly available in Zenodo at https://zenodo.org/, reference number https://doi.org/10.5281/zenodo.18865984.
