# Supplementary material for: Plant endemism of the Balkan high‐mountain fens: a community‐level synthesis and their climate change perspective
Source: Plant Biol (Stuttg). 2026 Aug 16;28(7):2393–409. doi: 10.1111/plb.70279 (PMC13750331; doi:10.1111/plb.70279)
Supplement: Supplementary file 1 [file PLB-28-2393-s001.pdf]

Table S1. List of Balkan endemic and sub-endemic vascular plant taxa narrowly associated with fen and spring habitats, including those reported in literature but not included into this paper because of their reconsideration or lack of data, and its comparison with the list of European wetland endemics by Hobohm & Bruchmann (2011). The table further includes the expert-based habitat characteristics, the list of formally described fen vegetation units (at lowest ranks; the symbol & is used when different ranks of different alliances are combined) for which the taxon is reported as diagnostic in at least one study, the elevation range based on distribution atlases compared with our data and notes with reasoning for their inclusion or exclusion and taxonomic notes.

No.	Taxon name	Broad habitat type	Habitat optimum	HOB	Vegetation units for which the taxon is reported as diagnostic	Elevation m (a.s.l.); our data in italics	Notes
**Taxa included into the analyses** (* refers to the taxa being used for initial relevé selection, the taxon indicating the *Narthecion scardici* alliance / the Relict mires of Mediterranean mountains EUNIS habitat)							
1.	*Cardamine acris* Griseb.	Springs, fens, tall herb vegetation, on different substrates	Spring	NO	*Pinguiculo balcanicae-Cardaminion acris*	*1705-2320*	Subendemic transgressing to Anatolia.
2.	*Carex castroviejoi* Luceño & Jim.Mejías	Fens and springs over serpentine substrates	Fen	NO	*Pinguiculo hirtiflorae-Cratoneuretum*	900-2000<br>*1634-2306*	Narrow-range endemic.
3.	* *Carex lazarei* Jac. Koopman, Niketić, Wieclaw & Govaerts	Alpine fens and snow beds, acidic	Fen	NO	*Narthecion scardici*,<br>*Pinguiculo-Plantagionion gentianoidis*	1000-1900<br>*1543-2452*	Syn.: *Carex bulgarica* (Domin) Lazare. Endemic.
4.	*Cirsium epiroticum* Petr.	Fens and wet (sub)alpine grasslands	Fen/Grass	NO	--	1000-2000<br>*1906-1985*	Ecology is insufficiently known. Endemic.
5.	** Cirsium heterotrichum* Pančić	(Sub)alpine fens and wet grasslands, on serpentine	Fen/Grass	NO	*Narthecion scardici* & *Cirsio heterotrichi-Caricetum nigrae*	1100-2500<br>*1463-2209*	Including *Cirsium halacsyi* Formánek (Pelister, Jablanitsa). Endemic.
6.	*Crocus pelistericus* Pulević	Fens, wet or semi-wet alpine turfs, on siliceous	Fen/Grass	NO	--	1600-2400<br>*1834-2232*	All records assigned as this taxon are from the Pelister and Kaimakchalan Mts where *C. pelistericus* s.s. occurs and frequently enters the fens. See the comment for *C. albanicus* Shuka *et al.* 2022 below.
7.	** Crocus veluchensis* Herb.	Fens, alpine grasslands, on different substrates	Fen/Grass	NO	*Cirsio heterotrichi-Caricetum nigrae*,<br>*Sphagno contorti-Primuletum exiguae*	1000-1700<br>*1834-2232*	Our data contain only *C. veluchensis* s.s. and all come from Bulgaria. The local endemics *C. jostii* Rukšāns & Zubov, *Crocus cvijicii* Košanin and *C. jablanicensis* N. Randj. & V. Randj. from North-Macedonia / Albania are not included and do not occur in fens.

No.	Taxon name	Broad habitat type	Habitat optimum	HOB	Vegetation units for which the taxon is reported as diagnostic	Elevation m (a.s.l.); our data in italics	Notes
8.	*Dactyloriza baumanniana* J. Hölzinger & Künkele	Fens, wet grasslands, on different substrates	Fen/Grass	NO	--	*1969-2192*	Only in two relevés; included only into species counts for wide-ranged endemics. Endemic.
9.	* *Dactylorhiza cordigera* (Fr.) Soó *sensu lato*	Fens and its margins, wet grasslands, springs, on different substrates	Fen/Grass	YES	*Dactylorhizo cordigerae-Eriophoretum latifolii*, *Cirsio heterotrichi-Caricetum nigrae*	800-2200 *1230-2370*	Including subspecies *cordigera* (= *siculorum* (Soó) Soó; subendemic transgressing to E Carpathians), *bosniaca* (Beck) Soó and *pindica* (B. Willing & E. Willing) H. Baumann & R. Lorenz.
10.	*Epilobium vernonicum* Snogerup	(Sub)alpine wet grasslands, springs and fens	Spring	NO	*Calthion palustris*, *Caricion fuscae* s.l.	1200-2000 *1757-2195*	Narrow-range endemic.
11.	* *Gentiana pyrenaica* L. auct.	Fens, (sub)alpine wet grasslands, snow beds	Fen/Grass	NO	*Primuletum exiguo-deori*	1700-2600 *2017-2417*	According to POWO (powo.science.kew.org), Bulgarian populations belong to Caucasian *Gentiana dshimilensis* K. Koch. Subendemic.
12.	* *Gentianella bulgarica* (Velen.) Holub	Fens, wet (sub)alpine grasslands, mostly in siliceous	Fen/Grass	NO	*Cirsio heterotrichi-Caricetum nigrae*, *Sphagno contorti-Primuletum exiguae*	800-2500 *1250-2600*	Endemic.
13.	* *Gymnadenia frivaldii* Hampe ex Griseb.	Permanently wet fens, on different substrates	Fen	NO	*Primulo exique-Caricetum echinatae*	1400-2600 *1250-2370*	Syn.: *Pseudorchis frivaldii* (Hampe ex Griseb.) P.F.Hunt. Subendemic transgressing to South Carpathians.
14.	*Heliosperma pusillum* subsp. *albanicum* (K.Malý) Niketić & Stevan.	Springs, calcareous spring fens, on different substrates	Spring	YES	*Cratoneurion commutati*, *Cratoneuro filicini-Calthion laetae*	1800-2200 *1500-2600*	Based on the results of Frajman & Oxelman (2007) we placed here also East-Balkan spring and fen populations of *H. pusillum* (Waldst. & Kit.) Rchb.
15.	*Juncus thomasii* Ten.	Fens, wet grasslands	Fen/Grass	YES	*Caricion fuscae* s.l., *Calthion palustris* & *Blysmo compressi-Eriophoretum latifoliae*	800-2000 *1250-2370*	Subendemic, transgressing to East / South Carpathians.
16.	* *Scorzoneroides rilaensis* (Hayek) Holub	Fens, (sub)alpine wet grasslands, snow beds, on siliceous	Fen/Grass	NO	*Primuletum exiguo-deori*	1900-2600 *1587-2395*	Syn.: *Leontodon rilaensis* Hayek. Subendemic transgressing to South Carpathians.
17.	* *Narthecium scardicum* Košanin	Fens; spring margins, mostly in serpentine and siliceous substrate	Fen	NO	*Narthecion scardici* (*Pinguiculo balcanicae-Narthecietum scardici*)	1300-2400 *1595-2306*	Narrow-range endemic.

No.	Taxon name	Broad habitat type	Habitat optimum	HOB	Vegetation units for which the taxon is reported as diagnostic	Elevation m (a.s.l.); our data in italics	Notes
18.	* *Pedicularis olympica* Boiss.	Fens, springs, on siliceous	Fen	YES	*Pinguiculo-Plantaginion gentianoidis*<br>*Pinguiculo balcanicae-Cardaminion acris*	1100-2300<br>*1757-2285*	Taxonomy follows Raus (1991) who considers *P. limnogena* A. Kern and *P. olympica* (described from Uludağ) as con-specific. Although the taxon (*sensu lato*) occurs in W Anatolia and the Carpathians, we classify it as narrow -range endemic.
19.	* *Pinguicula balcanica* Casper	(Sub)alpine fens and springs, rarely calcareous fens at lower elevations (Popovi livadi in the Pirin Mts), on different substrates	Fen	NO	*Primulo exique-Caricetum echinatae*,<br>*Pinguiculo balcanicae-Narthecietum scardici*,<br>*Sphagno contorti-Primuletum exiguae*,<br>*Blysmo compressi-Eriophoretum latifoliae*<br>& *Pinguiculo balcanicae-Cardaminion acris*	1400-2400<br>*1008-2460*	Endemic.
20.	*Pinguicula hirtiflora* Ten.	Calcareous springs, wet rocks, fens, on different substrates	Spring	NO	*Pinguiculo hirtiflorae-Cratoneuretum*	200-1500<br>*1073-2440*	Narrow-range endemic.
21.	* *Plantago gentianoides* Sibth. & Sm.	Fens, alpine wet grasslands, snow beds, siliceous and limestone	Fen/Grass	NO	*Pinguiculo-Plantagionion gentianoidis* (*Primuletum exiguo-deori*, *Carici nigrae-Plantaginetum gentianoidis*)	1500-2500<br>*1500-2600*	Subendemic transgressing to South Carpathians and Anatolyia.
22.	* *Primula deorum* Velen.	Alpine fens and wet grasslands	Fen	YES	*Primuletum exiguo-deori*	2000-2700<br>*2075-2418*	Narrow-range endemic (Rila Mts)
23.	* *Primula frondosa* subsp. *exigua* (Velen.) O. Schwarz	Alpine fens	Fen	YES	*Primuletum exiguo-deori*,<br>*Primulo exique-Caricetum echinatae*<br>*Sphagno contorti-Primuletum exiguae*	1600-2500<br>*1665-2452*	Narrow-range endemic (Rila, Pirin, Vitosha Mts)<br>Syn.: *Primula farinosa* subsp. *exigua* (Velen.) Hayek

No.	Taxon name	Broad habitat type	Habitat optimum	HOB	Vegetation units for which the taxon is reported as diagnostic	Elevation m (a.s.l.); our data in italics	Notes
24.	* *Silene asterias* Griseb.	Mountain fens, springs and, tall-herb and wet grasslands, mostly in siliceous	Fen/Grass	YES	*Narthecion scardici*, *Calthion palustris*, *Pinguiculo balcanicae-Cardaminion acris*	1600-2800 *1230-1997*	Endemic.
25.	* *Willemetia stipitata* subsp. *albanica* (Kümmerle & Jáv.) Kirschnerová	Fens, springs, tall herb vegetation, on different substrates	Fen/TH	YES	*Narthecion scardici* (*Willemetietum stipitatae*)	1600-2200 *1388-2320*	Endemic, missing in East Balkans.

Taxa reported as Balkan fen or spring endemics, but not finally included into the analyses

26.	*Cardamine barbareoides* Halácsy	Springs	Spring	NO	*Montio-Cardaminetea*	--	Insufficiently covered by our data. No relevé passed the selection rules. Never accompanied by another Balkan fen or spring endemics.
27.	*Carex dacica* Heuff.	Alpine fens and grasslands	Fen/Grass	NO	*Scheuchzerio palustris-Caricetea fuscae*	1700-2500	The name now spuriously applied to the widespread arctic-alpine taxon; the Balkan population may belong to formally non-described endemic taxon (see the note below the table) which was however not consistently identified in our relevés.
28.	*Carex macedonica* Bornmüller	Alpine wetlands	Fen	NO	*Scheuchzerio palustris-Caricetea fuscae*	--	Not accepted by Euro+Med checklist; see the note below the table
29.	*Cirsium tymphaeum* Hausskn.	Wet subalpine grasslands, tall herbs, margins of springs and fens, on different substrates	TH	YES	--	--	Optimum outside fens and springs. Insufficiently covered by our data. No relevé passed the selection rules. Never accompanied by other Balkan fen or spring endemics.
30.	*Crocus albanicus* Shuka *et al.* 2022	Fens, wet (sub)alpine grasslands on serpentinite	Fen/Grass	NO	--	1730-2170	The species described in a preprint paper (Shuka *et al.* 2022). Taxon is insufficiently covered by our data (one relevé) and was not hence included.
31.	*Epilobium gemmascens* C. A. Mey.	Gravel bars, springs, stream margins	--	NO	--	800-1600	Widespread range including Anatolyia, Caucasus and Iran. Clear optimum outside fens (gravel bars); we recorded it only in one relevé.

No.	Taxon name	Broad habitat type	Habitat optimum	HOB	Vegetation units for which the taxon is reported as diagnostic	Elevation m (a.s.l.); our data in italics	Notes
32.	*Geum rhodopaeum* Stoj. & Stef.	Wet grasslands, tall herb vegetation, fens	Grass	YES	*Calthion palustris*	--	Clear optimum outside fens and springs, only two relevés were classified as a fen.
33.	*Jacobaea pancicii* (Degen) Vladimir. & Raab-Straube	Tall-herb vegetation	TH	NO	--	1400-2000 *1853-2237*	Syn.: *Senecio pancicii* Degen. Recorded only in five fen relevés; mostly occured in tall-herb vegetation
34.	*Soldanella chrysosticta* Kress.	Alpine grasslands and shrublands, tall herb vegetation, fens	Grass	NO	--	*? 1634-2280*	Clear optimum outside fens. Insufficient distinguishing between *S. pindicola* Hausskn., *S. chrysosticta* and *S. rhodopaea* F. K. Mey in the data used (cf. Slovák *et al.* 2022)
35.	*Soldanella pindicola* Hausskn.	Alpine grasslands and shrublands,wet rocks, tall herb vegetation, fens, on serpentine and siliceous	Grass	YES	*Sphagno contorti-Primuletum exiguae* (erroneously; should be *S. chrysosticta*)	*? 1634-2280*	Clear optimum outside fens. Insufficient distinguishing between *S. pindicola*, *S. chrysosticta* and *S. rhodopaea* in the data used (cf. Slovák *et al.* 2022)

Explanations: Habitat optimum values: Grass = grasslands; TH = tall-herb habitats; HOB = Was the taxon included into the list of European wetland endemics by Hobohm & Bruchmann (2011)?. * = the taxon was used for the initial relevé selection (the taxon indicating the *Narthecion scardici* alliance / the Relict mires of Mediterranean mountains EUNIS habitat).

Comment to *Carex dacica* and *Carex macedonica*

Carex dacica Heuff. [= *C. bigelowii* subsp. *dacica* (Heuff.) T. V. Egor.] is recently considered as a widespread taxon from the *Carex bigelowii* group with a large arctic-alpine distribution range including not only Central Europe, but also Norway and Iceland (Westergaard *et al.* 2021). However, the original description seems to be incorrectly applied (Radomír Řepka, personal information) and, consequently, plants growing in many high-mountain wetlands of the Southern Carpathians and the Balkans are referred to as *Carex dacica* auct. non Heuffel (or as *C. macedonica*), being considered subordinate to *Carex nigra* instead of *C. bigelowii* Schwein. Nevertheless, a set of morphological features such as smaller tufts with shorter rhizome, dark red basal sheaths, slender stems, narrow green leaves, contracted inflorescences with dark spikes and a short lowest bract, suggests that these South Carpathian-Balkan plants may belong to a separate taxon from the *Carex nigra* group (provisionally named *C. balcanica*), well delimited from other taxa by both the morphology and the distribution (Radomír Řepka, personal information). The recent phylogenetic study of Valdéz-Florido *et al.* (2025) suggests that southern Balkan high-mountain populations of *C. nigra* may belong to subsp. *transcaucasica* (T.V.Egorova) Jim.Mejías, G.E.Rodr.-Pal., Amini Rad & Martín-Bravo, which was previously known from Anatolyia and Caucasus. However, this assumption is based on a single site from the Balkans (Lurë in Albania). As the Balkan high-mountain populations of *C. nigra* agg. show considerable morphological differences against *C. *transcaucasica* (Radomír Řepka, personal information), the occurrence of the latter taxon in the Balkans is still unclear.

The list of non-endemic vascular plant fen taxa

Agrostis canina L., *Allium schoenoprasum* L., *Blysmus compressus* (L). Link, *Cardamine rivularis* Schur., *Carex buxbaumii* Wahlenb., *Carex canescens* L., *Carex davalliana* Sm., *Carex demissa* Hornem., *Carex echinata* Murray, *Carex flava* L., *Carex frigida* All., *Carex hostiana* DC., *Carex lasiocarpa* Ehrh., *Carex lepidocarpa* Tausch, *Carex limosa* L., *Carex nigra* agg., *Carex panicea* L., *Carex pauciflora* Lightf., *Carex rostrata* Stokes, *Carex viridula* Michx., *Comarum palustre* L., *Dactylorhiza incarnata* (L.) Soó, *Dactylorhiza maculata* agg., *Drosera rotundifolia* L., *Eleocharis quinqueflora* (Hartmann) O. Schwarz, *Epilobium palustre* L., *Epipactis palustris* (L.) Crantz, *Equisetum variegatum* F. Weber & D. Mohr, *Eriophorum angustifolium* Honck., *Eriophorum gracile* Roth, *Eriophorum latifolium* Hoppe, *Eriophorum vaginatum* L., *Juncus alpinoarticulatus* Chaix, *Juncus articulatus* L., *Juncus bulbosus* L., *Juncus filiformis* L., *Juncus triglumis* L., *Luzula sudetica* (Willd.) Schult., *Menyanthes trifoliata* L., *Parnassia palustris* L., *Pedicularis palustris* L., *Pedicularis verticillata* L., *Pinguicula vulgaris* L., *Polygala amarella* Crantz, *Ranunculus flammula* L., *Salix lapponum* L., *Sesleria uliginosa* Opiz, *Swertia perennis* L., *Thalictrum alpinum* L., *Trichophorum cespitosum* (L.) Hartm., *Triglochin palustris* L., *Utricularia minor* L., *Utricularia ochroleuca* R. W. Hartm., *Vaccinium oxycoccos* L., *Vaccinium uliginosum* L., *Valeriana dioica* subsp. *simplicifolia* (Rchb.) Nyman, *Viola palustris* L.

The list of non-endemic bryophyte fen taxa

Aulacomnium palustre (Hedw.) Schwägr., *Calliergon giganteum* (Schimp.) Kindb., *Calypogeia sphagnicola* (Arnell & J. Perss.) Warnst. & Loeske, *Campylium stellatum* (Hedw.) Lange & C. E. O. Jensen, *Dicranum bonjeanii* De Not., *Dicranum leioneuron* Kindb., *Drepanocladus trifarius* (F. Weber & D. Mohr) Broth., *Fissidens adianthoides* Hedw., *Fissidens osmundoides* Hedw., *Gymnocolea inflata* (Huds.) Dumort., *Hamatocaulis vernicosus* (Mitt.) Hedenäs, *Meesia longiseta* Hedw., *Meesia triquetra* ([L. ex] Jolycl.) Ångström, *Meesia uliginosa* Hedw., *Mesoptychia bantriensis* (Hook.) L.Söderstr. & Váňa, *Mylia anomala* (Hook.) Gray, *Paludella squarrosa* (Hedw.) Brid., *Philonotis caespitosa* Jur., *Philonotis calcarea* (Bruch & Schimp.) Schimp., *Philonotis fontana* (Hedw.) Brid., *Philonotis tomentella* Molendo, *Polytrichum strictum* [Menzies ex] Brid., *Ptychostomum pseudotriquetrum* (Hedw.) J. R. Spence & H. P. Ramsay ex Holyoak & N. Pedersen, *Riccardia multifida* (L.) Gray, *Sarmentypnum exannulatum* (Schimp.) Hedenäs, *Sarmentypnum sarmentosum* (Wahlenb.) Tuom. & T.J.Kop., *Scorpidium cossonii* (Schimp.) Hedenäs / *Scorpidium revolvens* (Sw. ex Anon.) Rubers, *Scorpidium scorpioides* (Hedw.) Limpr., *Sphagnum angustifolium* (C. E. O. Jensen ex Russow) C. E. O. Jensen, *Sphagnum auriculatum Schimp.*, *Sphagnum capillifolium* (Ehrh.) Hedw., *Sphagnum centrale* C. E. O. Jensen, *Sphagnum compactum* Lam. & DC., *Sphagnum contortum* Schultz, *Sphagnum cuspidatum* Ehrh. ex Hoffm., *Sphagnum fallax* (H. Klinggr.) H. Klinggr., *Sphagnum flexuosum* Dozy & Molk., *Sphagnum inundatum* Russow, *Sphagnum magellanicum* Brid., *Sphagnum molle* Sull. (L. Melovski data, 1 occurrence), *Sphagnum palustre* L., *Sphagnum papillosum* Lindb., *Sphagnum platyphyllum* (Lindb. ex Braithw.) Sull. ex Warnst., *Sphagnum rubellum* Wilson, *Sphagnum russowii* Warnst., *Sphagnum squarrosum* Crome, *Sphagnum subnitens* Russow & Warnst., *Sphagnum subsecundum* Nees, *Sphagnum teres* (Schimp.) Ångström, *Sphagnum warnstorfii* Russow, *Straminergon stramineum* (Dicks. ex Brid.) Hedenäs, *Tayloria lingulata* (Dicks.) Lindb, *Tomentypnum nitens* (Hedw.) Loeske

References

Frajman B., Oxelman B. (2007) Reticulate phylogenetics and phytogeographical structure of *Heliosperma* (Sileneae, Caryophyllaceae) inferred from chloroplast and nuclear DNA sequences. *Molecular Phylogenetics and Evolution*, **43**, 140–155. https://doi.org/10.1016/j.ympev.2006.11.003

Hobohm C., Bruchmann I. (2011) Are there endemic vascular plants in wet habitats of Europe? *Transylvanian Review of Systematical and Ecological Research*, **12**, 1–14.

Raus T. (1991) *Pedicularis* L. In: Strid A., Tan K. (Eds), *Mountain Flora of Greece*, Vol. 2. University Press, Edinburgh, UK.

Shuka D., Kerndorff H., Waminal N., Ranđelovič V., Harpke D. (2022) Two new species of *Crocus* ser. *Scardici* from the Western Balkans. *bioRxiv*, preprint. https://doi.org/10.1101/2022.10.27.514018

Slovák M., Melichárková A., Štubňová E.G., Kučera J., Mandáková T., Smyčka J., Lavergne S., Passalacqua N.G., Vďačný P., Paun O. (2022) Pervasive introgression during rapid diversification of the European mountain genus *Soldanella* (L.) (Primulaceae). *Systematic Biology*, **72**, 491–504. https://doi.org/10.1093/sysbio/syac071

Valdés-Florido A., Gómez I., Escudero M., Maguilla E., Luceño M., Jiménez-Mejías P., Benítez-Benítez C. (2024) Genomic and ecological insights into phylogeography: the complex history of the widespread herb *Carex nigra* (Cyperaceae) revisited. *Alpine Botany*, **135**, 19–31. https://doi.org/10.1007/s00035-024-00322-y

Westergaard K.B., Kyrkjeeide M.O., Brandrud M.K. (2021) Using genomics to guide seed-sourcing at the right taxonomical level for ecological restoration projects: the complex case of *Carex bigelowii* s. lat. in Norway. *Ecology and Evolution*, **11**, 17117–17131. https://doi.org/10.1002/ece3.8350
