# Supplementary material for: Plant endemism of the Balkan high‐mountain fens: a community‐level synthesis and their climate change perspective
Source: Plant Biol (Stuttg). 2026 Aug 16;28(7):2393–409. doi: 10.1111/plb.70279 (PMC13750331; doi:10.1111/plb.70279)
Supplement: Supplementary file 2 [file PLB-28-2393-s002.pdf]

Appendix S1

Extended materials and methods

(for an article *Plant endemism of the Balkan high-mountain fens: a community-level synthesis and their climate change perspective*, currently under review in *Biogeosciences,* text in grey font corresponds to the main text; text in black font presents the extended content)

Study area and its climate 1
The list of Balkan endemics, selection of vegetation-plot records and taxonomic harmonisation 2
Addressing taxonomic uncertainties 3
Classification of endemic taxa into two groups 3
Environmental variables 3
Mapping the hotspots 4
Data analyses 5
Pattern across vegetation types 5
Pattern across the past and present environment 5
Pattern across regions 6
References 6

Study area and its climate

The study area comprises the high mountains of the Balkan Peninsula and the adjacent Southern Carpathians (Romania). The area is shared by nine countries: Albania, Bosnia and Herzegovina, Bulgaria, Greece, Kosovo, Montenegro, North Macedonia, Romania, and Serbia. It includes major mountain ranges of southeastern Europe, such as the Southern Carpathians, the Central Balkan range, the Rila-Rhodope massif, the Scardo-Pindus range, the Dinarids, and others (Fig. 1). Only those parts of these ranges where high-mountain fens with Balkan endemics occur were included. From a plant-geographical perspective, the region belongs to the Illyrian and Balkanic regions (divided into West-Moesian, East-Moesian, and Scardo-Pindus) (Horvat *et al.* 1974). The individual parts of the study area differ in current climate, palaeoclimate, topography, and edaphic factors, the effects of which were tested in this study. The high-resolution climate map of Botti (2018) shows that Balkan high mountains represent isolated cold-climate islands. According to the updated Köppen climate classification (Beck *et al.* 2023), most of these mountain islands belong to the Dfb zone (cold with no dry season and warm summers), but the southern and southeastern parts of the study area transition to or belong to the Dbb zone (cold with dry warm summer). During the Quaternary, isolated mountain glaciers existed during the LGM between 29,000 and 19,000 BP (Kuhleman *et al.* 2009; Tonkov 2021; Hughes *et al.* 2022). Between them, alpine tundra locally developed (Tonkov 2021). The mountain steppe was the dominant habitat throughout the glacial periods, as indicated by Artemisia-, Poaceae-, Ephedra-, and Juniperus-type pollen, together with additional grassland indicators (Tonkov 2021). The relatively dry climate of the Balkan high mountains persisted throughout the Late Pleniglacial (Late High Glacial) and the early Holocene (Wright *et al.* 2003). According to Divíšek *et al.* (2021), the Balkans were among the climatically most suitable areas for steppes in Europe during both the LGM and the Late Pleniglacial. This late Pleistocene climate history supports the hypothesis that fen endemism is higher in regions that have continuously retained tundra conditions, at least locally, within landscapes otherwise dominated by dry steppe or forest-supporting climates. Considering the magnitude of climate variability from the LGM to the present, which well represents Quaternary climate variability (Sandel *et al.* 2017), our focus on the last 21,000 years may provide reliable insights into the past drivers of fen endemism.

The list of Balkan endemics, selection of vegetation-plot records and taxonomic harmonisation

As the first step, we assembled the candidate list of Balkan endemics whose occurrence is at least partially associated with fens (Tab. S1, see Fig. S1 for photos). The plant nomenclature was harmonised using Euro+Med PlantBase (Euro+Med 2006+), with a few exceptions described in Table S1. As the next step, we reduced the list to taxa whose optimum is centred on fens or springs, rather than on wet grasslands or wetland tall herb vegetation. This rule was assessed arbitrarily based on the field experience of all authors and on published vegetation data from regions where not only fens, but also wet grasslands and tall-herb stands were sampled with the same intensity (e.g. Hájková *et al.* 2008 and Szokala *et al.* 2024 for Bulgaria). The excluded taxa had been found only rarely in our fen-vegetation dataset. In the next step, we selected the vegetation-plot records (hereinafter referred to as *relevés*) for the analysis. For this selection, we defined the subset of 16 taxa that had been previously reported as diagnostic for the vegetation types and habitats defined by Balkan (sub)endemics, i.e. the *Narthecion scardici* alliance (Peterka *et al.* 2017) and the Relict mires of Mediterranean mountains (Chytrý *et al.* 2020), see Table S1. The resulting pool of relevés comprises our original data and a limited number of relevés from other authors (Tab. S2).

Data were collected in the summer months (June to August) during the period 2001 to 2025 across all elevations. For this study we selected the relevés in which any of these 16 taxa occur with any cover code. This step excluded sites below ~ 1000 m a.s.l., thereby focusing the analysis narrowly on the mountains. The pool of relevés for this selection consisted of our original data, unpublished data from L. Melovski from Šar Planina (N Macedonia), and a limited number of relevés from the literature (Tab. S2). The dataset was assembled during field research focused on sedge-moss spring and fen vegetation; other vegetation types, such as matgrass pasture and wet heath, were omitted. The applied selection rule resulted in 410 selected relevés, out of which 376 relevés contained species composition of the moss layer. The taxa were classified into narrow-range endemics (7 taxa), wide-range endemics (18 taxa), vascular plants specialised to fens (57 taxa), bryophytes specialised to fens (53 taxa) and other taxa (415), following the assignments of Mucina *et al.* (2016), Peterka *et al.* (2017), Horsáková *et al.* (2018) and Hájek *et al.* (2022). See Table S1 for species lists. For further species-level analyses, we kept only those endemic taxa which occurred in at least 8 vegetation-plot records (i.e. in at least 2% of relevés).

The most relevés come from the region of Rila and Pirin Mts and neighbouring areas (Bulgaria), followed by the mountain range formed by the Šar, Korab and Jablanitsa Mts (North Macedonia, Kosovo, Albania) and distantly by the Prokletije/Accursed Mountains (Montenegro, Kosovo, Albania), Stara Planina Mts (Bulgaria, Republic of Serbia), serpentinite mountains of southwestern part of the study area (Valamara, Lurë, Gjergjevica, Mashkullora of Panarit in Albania and Mavrovouni and Smolikas in Greece) and the Voras (Kaimakchalan, Nidže) Mts (Greece, North Macedonia). Other mountain ranges are less represented, mainly because the area or number of sites harbouring Balkan fen endemics is smaller there (Vitosha Mts, Pelister Mts, Zelengora Mts, Bjelasica Mts, Komovi Mts, Kopaonik Mts, Varnous Mts, Osogovo Mts, Solunska Glava Mts, Vlasina Plain and mountains of Southern Carpathians such as Retezat, Lotrului, Făgăraș and Cindrel Mts).

Vegetation plot data were collected using the Braun-Blanquet approach with the nine-grade cover scale (Westhoff & van der Maarel 1978) during the summer months (June to August) from 2001 to 2025 across all elevations, but the analysed dataset was restricted during data filtering, resulting in a minimum elevation of approximately 1000 m (App. S1). Both the original nine-grade scale and its conversion to mid-point values (according to the JUICE software, Tichý 2002) were used in the individual analyses. Although most plots were sampled using the same protocol with a standard plot size of 16 m^2, the analysed dataset includes plots of different sizes (2–100 m^2). Smaller plot sizes were used when the spring or the fen containing Balkan endemics occurred only fragmentarily, which prevented larger plot sizes. Larger plot sizes primarily come from the database or unpublished data of L. Melovski. Plot size was included as a predictor in subsequent analyses. The motivation for treating plot size as an independent factor was to test for potential differences in the plot-size effect across species groups.

Addressing taxonomic uncertainties

The classification of some fen taxa in the Balkans as endemics, or the distinction between narrow-range and wide-range endemics, is complicated by unresolved taxonomy. This problem applies to *Pedicularis olympica* Boiss., *Gentiana pyrenaica* L. auct. — both taxa are included, yet with uncertain classification as narrow- or wide-ranged in the literature — and for *Soldanella pindicola* Hausskn*., S. chrysosticta* Kress. and *Crocus albanicus* Shuka *et al.* 2022*,* where taxonomic know-ledge had evolved during our long-term research, and these taxa were not finally included because of both the insufficient coverage by our data and the substantial niche extension outside fens and springs (Tab. S1). The taxa *Cirsium halacsyi* Formánek and *Crocus jostii* Rukšāns & Zu-bov are not accepted by the Euro+Med Plantbase (Euro+Med, 2006+) and are therefore merged with the accepted taxa. The most complicated issue concerns the *Carex* sect. *Phacocystis* Dumort*.,* which may include the potentially endemic taxon allied to *C. nigra* (L.) Reichard, is sometimes referred to as *C. dacica* Heuff. — the name recently spuriously applied to a widespread arctic-alpine taxon from the *C. bigelowii* group — or as *C. macedonica* Bornmüller. Because of unresolved taxonomy and the inability to distinguish between individual taxa in many relevés, we merged C. nigra and C. dacica/*macedonica* auct. into a single aggregate (*Carex nigra* agg.). See the detailed reasoning in comments on Table S1.

Classification of endemic taxa into two groups

The selection rules resulted in 25 Balkan fen species being examined in detail. Out of them, 7 were classified as narrow-ranged Balkan fen endemics and 18 as wide-ranged Balkan fen endemics (Tab. S1). This classification was done arbitrarily, with the group of narrow-ranged endemics containing two taxa endemic to Bulgarian high mountains (*Primula deorum* Velen. as a stenoendemic of the Rila Mts and *P. frondosa* subs. *exigua* (Velen.) O. Schwartz as an endemic of the Rila, Pirin, Vitosha and a part of Stara Planina Mts), three taxa endemic to southwestern part of the study area not reaching eastern half neither northwestern margin of the study area (*Narthecium scardicum* Košanin*, Carex castroviejoi* Luceño & Jim.Mejías, *Pinguicula hirtiflora* Ten.) and two taxa restricted to central part of the study area across Greek-North Macedonia and Albania borders (*Epilobium vernonicum* Snogerup, *Pedicularis olympica* Boiss.). The assignment of *P. olympica* mirrors the current taxonomic scheme, which merges Balkan populations previously assigned as *P. limnogena* A. Kern. (described from the Apuseni Mts in the Carpathians) with *P. olympica* (see Tab. S1 for details). The isolated occurrence of *P. olympica* in the West Anatolia (Turkey) was not considered as an obstacle for classifying the taxon as a narrow-ranged endemic in the context of the entire study area, as the taxon is missing in the substantial parts of the study area where other Balkan endemics are concentrated (eastern, northern and western parts). Another arbitrary decision is placing three narrow-range taxa with not sufficiently known ecology and distribution due to taxonomic uncertainties into the group of wide-range endemics (*Cirsium epiroticum* Petr*., Heliosperma pusillum* subsp. *albanicum* (K.Malý) Niketić & Stevan*., Crocus pelistericus* Pulević). The remaining 14 wide-range endemics include *Pinguicula balcanica* Casper, *Gymnadenia frivaldii* Hampe ex Griseb., *Dactylorhiza cordigera* (Fr.) Soó sensu lato (mostly occurring as subsp. *Bosniaca* (Beck) Soó, see Tab. S1), *Willemetia stipitata* subsp. *albanica* (Kümmerle & Jáv.) Kirschnerová, *Plantago gentianoides* Sibth. & Sm*., Carex lazarei lazarei* Jac. Koopman, Niketić, Wieclaw & Govaerts (Syn: *C. bulgarica* (Domin) Lazare)*, Cardamine acris* Griseb*., Juncus thomasii* Ten.*, Gentianella bulgarica* (Velen.) Holub*, Cirsium heterotrichum* Pančić (including *Cirsium halacsyi* Formánek not accepted by Euro+Med PlantBase (Euro+Med 2006+)), *Gentiana pyrenaica* L. auct. (Syn: *G. dhsmiliensis* K. Koch), *Crocus veluchensis* Herb.*, Leontodon rilaensis* Hayek and *Silene asterias* Griseb.

Environmental variables

We supplemented individual relevés with the direct field measurements of water pH and conductivity (EC). For the 132 and 147 relevés, respectively (primarily data from external authors), we imputed missing values using the Moss method (Tichý *et al.* 2010a). To account for their synergistic or mutually substitutive effects, water pH and EC were combined into a single variable (adjusted pH) as described by Plesková *et al.* (2016). To control for potential differences in hydrology, we also calculated the arithmetic mean of ecological indicator values (EIV) for water level,

weighted by inverse species tolerances (niche width), using European-scale values for both vascular plants and bryophytes (Hájek *et al.* 2020).

We further extracted, for the location of each relevé, the current and the past macroclimate parameters including mean annual temperature (bio1), mean temperature of warmest quarter (bio10; summer temperature), mean temperature of the coldest quarter (bio11; winter temperature), annual precipitation (bio12), precipitation of warmest quarter (bio18; summer precipitation) and precipitation of coldest quarter (bio19; winter precipitation). These variables were obtained at a spatial resolution of 30 arc seconds from the CHELSA Timeseries and CHELSAcruts datasets as 50-year averages for 1940–1989 AD (Karger *et al.* 2017), and from downscaled and debiased paleoclimate simulations of the Community Climate System Model ver. 3 (CCSM3; Divíšek 2021) for past periods. Based on paleoclimate data spanning the last 21,000 years at a 100-year temporal resolution, we calculated the maximum positive and negative deviations (absolute values) from the recent value for each of the above-mentioned variables (Fig. A) and the climate stability index.

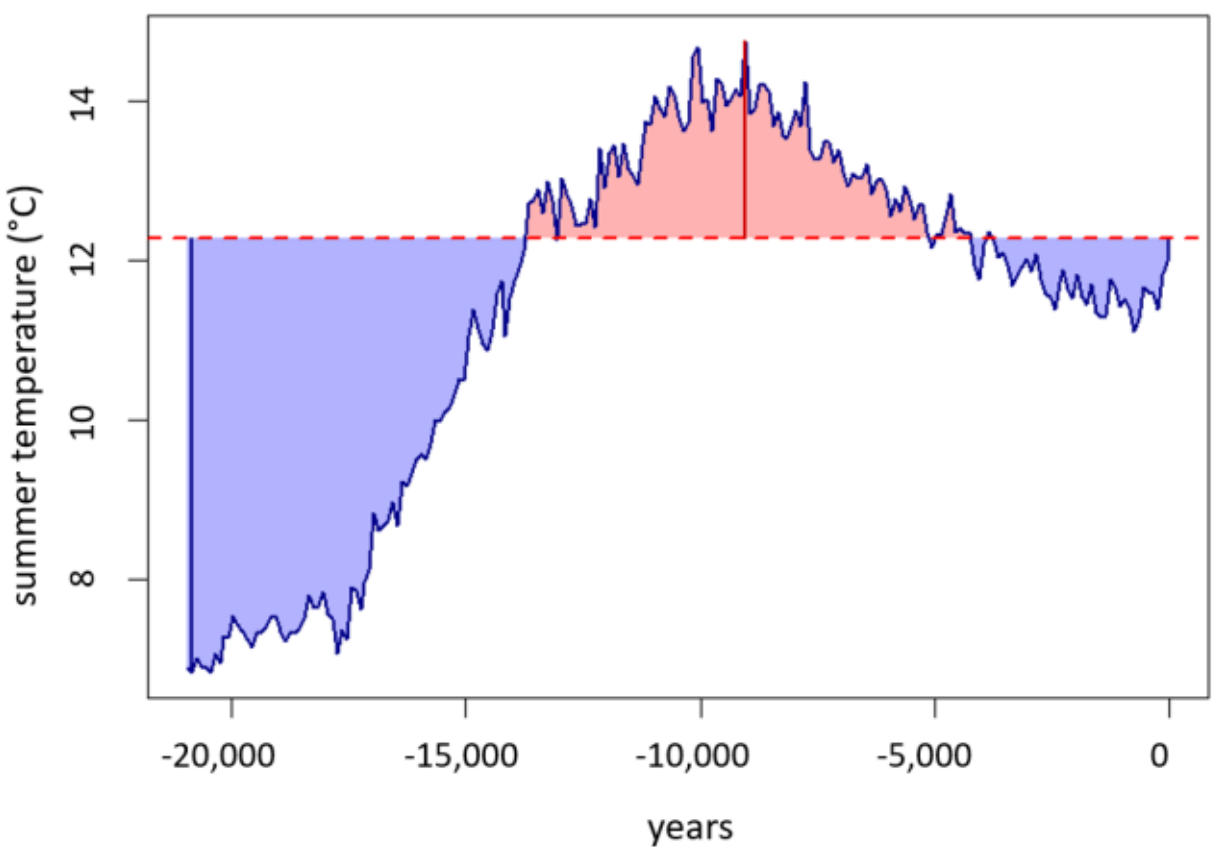

Fig. A. Graph illustrating the example of how the minimum and maximum deviations from the recent value were extracted (summer temperature in last 20,000 years)

For precipitation variables, the deviation was expressed as a percentage of the recent value. The index of climate stability was calculated as follows: for bio10 and bio11, an average absolute deviation from the recent value was calculated. For bio18 and bio19, we calculated the average percentage deviation from the recent value. Following Herrando-Moraira *et al.* (2022), each of these deviations was then normalised to values between 0 and 1, with 0 rep-resenting completely stable areas and 1 the most unstable ones. Overall climate stability since the LGM was then calculated for each relevé as 1 minus the mean of the normalised deviations. Higher values of the index thus indicate higher climate stability.

We further used a digital elevation model at a 90 m resolution (Jarvis *et al.* 2008) and calculated elevation (m a.s.l.) and the Terrain Ruggedness Index (TRI) for each site. We also created 5 km and 10 km buffer zones around each relevé, and within these zones we calculated minimum, mean, and maximum elevation and TRI, as well as elevation and TRI ranges. Finally, within these buffer zones, we also calculated the area at elevations above 1,700 m, with the premise that a larger area indicates a more massive mountain range, buffering the effects of short-term climate fluctuations and, hence, showing more stable cold-climate conditions recently and more opportunities to find cold-microclimate refugia during any warm period.

Mapping the hotspots

We identified hotspots of community-scale diversity in Balkan fen endemics by applying kernel density estimation (KDE) to point-based relevé data using the Heatmap (KDE) tool in QGIS 3.34 (QGIS.org 2026). A fixed search radius (bandwidth) of 50 km was applied uniformly across all plant groups to ensure comparability of spatial patterns. This relatively wide radius was chosen to produce generalised results. Each relevé was weighted by the number of taxa within the respective plant group, using the corresponding attribute field as a weight parameter in the QGIS Heatmap tool. The output was visualised using a continuous colour ramp representing gradients from low to high diversity, with each panel displayed using an independent colour scale.

Data analyses

Pattern across vegetation types

For the general classification of vegetation into major vegetation types, we used the modified Twinspan algorithm (Roleček *et al.* 2009). Only 376 relevés with identified bryophytes were included. As the study area encompasses a long edaphic gradient of pH and calcium to which fen vegetation usually responds (Hájek *et al.* 2006), but other, context-dependent gradients may also be important for the Balkan fen vegetation in the different parts of the major pH gradient (Hájková *et al.* 2006), we decided on Twinspan as a hierarchical classification method. Another advantage of Twinspan in our context is its divisive nature, which first generalises compositional variation by finding and then splitting the major gradient. This algorithm hence suppresses the effects of local peculiarities, for example, caused by contact vegetation types or specific management, as well as observer effects that may arise when using literature data. The algorithm was run on presence/absence data, with cover-abundance values partially retained by cloning the taxon if its cover value exceeded the threshold (*pseudo-species cut-levels* according to Hill *et al.* 1975). Thresholds of 5% (2m) and 25% (3) were adopted, above which the taxon was doubled or tripled, respectively. We set the number of clusters using the Optimclass Method (Tichý *et al.* 2010b), with a preference for a relatively low number of clusters, as vegetation classification was not the ultimate aim of the study. The diagnostic taxa for the clusters were identified by a combination of the significance of Fisher's exact test of the taxon's association with the cluster and species frequency. We present the results of Fisher's exact test for all Balkan endemics showing at least 20% frequency in a cluster.

Pattern across the past and present environment

For the 375 relevés (one sample from Melovski's dataset was deleted due to wrong coordinates revealed during data processing) we simplified the complex space of 32 environmental variables into a two-dimensional ordination space using Principal Component Analysis, retaining the first two axes. The variables were standardised and centred to ensure comparability. In the resulting ordination space, we show the community-scale richness of wider- and narrow-ranged Balkan endemics, or the presence of individual endemic taxa, using different symbol sizes and isolines of Generalised Additive Models with complexity set by the Akaike Information Criterion.

The effects of individual explanatory variables on the species composition of Balkan endemics (both groups merged; *Cirsium epiroticum* excluded due to outlier effects) at the community level were tested using canonical correspondence analysis (CCA) with a Monte Carlo test. The mid-point percentage values were log-transformed. Forward selection was stopped when the contribution of a newly added variable fell below 5%, as more liberal thresholds produced a strong arch effect.

For each group (narrow-range endemics, wide-range endemics, all endemics, non-endemic fen vascular plants, non-endemic fen bryophytes and other, i.e. matrix-derived taxa), we tested the Spearman rank correlations between richness and environmental variables. Resulting p-values were adjusted using the Bonferroni correction to address multiple testing. We then tested how the results change when controlled for multicollinearity and spatial autocorrelation using the spatial simultaneous autoregressive error model (SAR_{err}). For SAR_{err}, we selected environ-mental variables that significantly correlated with any richness value. If the Spearman correlation between any two selected variables exceeded 0.7, we removed the variable with the lower correlation with taxa richness. The spatial weight matrix used by SAR_{err} was constructed from Moran's correlograms calculated for each group's taxonomic richness using the correlog function in the *pgirmess* R package. The distance at which spatial autocorrelation decreased to zero was recorded, and plots within this distance were considered neighbours when constructing the spatial weight matrix. We interpreted the results of both Spearman correlations and SAR_{err} (the latter not shown in detail) in the light of the fact that climate, especially palaeoclimate variables modelled at coarse spatial scales, is inherently shared across large areas and therefore strongly spatially autocorrelated, leading to penalisation in the SAR_{err} models.

Pattern across regions

We further classified individual relevés by their location in a pre-defined region. We divided the dataset into 11 regions (Tab. S4), corresponding to individual mountain ranges or groups of neighbouring mountain ranges forming one mountain chain (Love *et al.* 2023) that share similar environmental conditions; the latter solution was used when the individual mountain range was underrepresented. For each of the 11 regions, we calculated the percentage frequency of each Balkan endemic taxon (across both groups). The resulting taxon-by-region matrix was subjected to the Principal Coordinate Analysis (PCoA) with the Hellinger distance as a dissimilarity measure. Note that the distance is applied to non-transformed compositional proportions, depending on the regional boundaries defined in Table S2.

The resulting biplot describes significant gradients in the representation of individual endemic taxa in fen communities at the scale of individual Balkan regions.

PCA, CCA, and PCoA were analysed using Canoco 5 software (ter Braak & Šmilauer 2012). TWINSPAN and diagnostic taxa delimitation were performed in JUICE 7.1.25 software (Tichý 2002). Other statistical analyses were conducted using the R 4.2.1 package (R Core Team 2022).

References

Beck H.E., McVicar T.R., Vergopolan N., Berg A., Lutsko J.N., Dufour A., Zeng Z., Jiang X., van Dijk A.I.J.M., Miralles D.G. (2023) High-resolution (1 km) Köppen-Geiger maps for 1901–2099 based on constrained CMIP6 projections. *Scientific Data*, **10**, 724. https://doi.org/10.1038/s41597-023-02549-6

Botti D. (2018) A phytoclimatic map of Europe. *Cybergeo*, **867**. https://doi.org/10.4000/cybergeo.29495

Chytrý M., Tichý L., Hennekens S.M., Knollová I., Janssen J.A.M., Rodwell J.S., Peterka T., Marcenò C., Landucci F., Danihelka J., Hájek M. (2020) EUNIS Habitat Classification: Expert system, characteristic species combinations and distribution maps of European habitats. *Applied Vegetation Science*, **23**, 648–675. https://doi.org/10.1111/avsc.12519

Divíšek J. (2021) High-resolution BIOCLIM and ENVIREM grids for Europe in consecutive 100-year bins spanning the last 21,000 years (1.0). Zenodo, Geneva, Switzerland [dataset]. Available at: https://doi.org/10.5281/zenodo.5119958

Euro+Med (2006 onwards) Euro+Med PlantBase – the information resource for Euro-Mediterranean plant diversity. Available at: http://www.europlusmed.org (accessed 12 December 2025).

Hájek M., Horsák M., Hájková P., Dítě D. (2006) Habitat diversity of central European fens in relation to environmental gradients and an effort to standardise fen terminology in ecological studies. *Perspectives in Plant Ecology, Evolution and Systematics*, **8**, 97–114. https://doi.org/10.1016/j.ppees.2006.08.002

Hájek M., Dítě D., Horsáková V., Mikulášková E., Peterka T., Navrátilová J., Jiménez-Alfaro B., Hájková P., Tichý L., Horsák M. (2020) Towards the pan-European bioindication system: Assessing and testing updated hydrological indicator values for vascular plants and bryophytes in mires. *Ecological Indicators*, **116**, 106527. https://doi.org/10.1016/j.ecolind.2020.106527

Hájek M., Těšitel J., Tahvanainen T., Peterka T., Jiménez-Alfaro B., Jansen F., Pérez-Haase A., Garbolino E., Carbognani M., Kolari T.H.M., Hájková P., Jandt U., Aunina L., Pawlikowski P., Ivchenko T., Tomaselli M., Tichý L., Dítě D., Plesková Z., Mikulášková E. (2022) Rising temperature modulates pH niches of fen species. *Global Change Biology*, **28**, 1023–1037. https://doi.org/10.1111/gcb.15980

Hájková P., Hájek M., Apostolova I. (2006) Diversity of wetland vegetation in the Bulgarian high mountains, main gradients and context-dependence of the pH role. *Plant Ecology*, **184**, 111–130. https://doi.org/10.1007/s11258-005-9056-5

Hájková P., Hájek M., Apostolova I., Zelený D., Dítě D. (2008) Shifts in the ecological behaviour of plant species between two distant regions: evidence from the base richness gradient in mires. *Journal of Biogeography*, **35**, 282–294. https://doi.org/10.1111/j.1365-2699.2007.01793.x

Herrando-Moraira S., Nualart N., Galbany-Casals M., Garcia-Jacas N., Ohashi H., Matsui T., Susanna A., Tang C.Q., López-Pujol J. (2022) Climate Stability Index maps, a global high resolution cartography of climate stability from Pliocene to 2100. *Scientific Data*, **9**, 48. https://doi.org/10.1038/s41597-022-01144-5

Hill M.O., Bunce R.G.H., Shaw M.W. (1975) Indicator species analysis, a divisive polythetic method of classification and its application to a survey of native pinewoods in Scotland. *Journal of Ecology*, **63**, 597–613. https://doi.org/10.2307/2258738

Horsáková V., Hájek M., Hájková P., Dítě D., Horsák M. (2018) Principal factors controlling the species richness of European fens differ between habitat specialists and matrix-derived species. *Diversity and Distributions*, **24**, 742–754. https://doi.org/10.1111/ddi.12718

Horvat I., Glavač V., Ellenberg H. (1974) *Vegetation Südosteuropas*. G. Fischer-Verlag, Stuttgart, Germany: 768 pp.

Hughes P.D., Allard J.L., Woodward J.C. (2022) The Balkans: glacial landforms from the Last Glacial Maximum. In: Palacios D., Hughes P.D., García-Ruiz J.M., Andrés N. (Eds), *European Glacial Landscapes, Maximum Extent of Glaciations*. Elsevier, Amsterdam, Netherlands: 487–495. https://doi.org/10.1016/B978-0-12-823498-3.00058-3

Karger D.N., Conrad O., Böhner J., Kawohl T., Kreft H., Soria-Auza R.W., Zimmermann N.E., Linder H.P., Kessler M. (2017) Climatologies at high resolution for the earth's land surface areas. *Scientific Data*, **4**, 170122. https://doi.org/10.1038/sdata.2017.122

Kuhlemann J., Milivojević M., Krumrei I., Kubik P.W. (2009) Last glaciation of the Šara range (Balkan peninsula): increasing dryness from the LGM to the Holocene. *Australian Journal of Earth Sciences*, **102**, 146–158.

Love S.J., Schweitzer J.A., Woolbright S.A., Bailey J.K. (2023) Sky Islands are a global tool for predicting the ecological and evolutionary consequences of climate change. *Annual Review of Ecology, Evolution, and Systematics*, **54**, 219–236. https://doi.org/10.1146/annurev-ecolsys-102221-050029

Mucina L., Bültmann H., Dierßen K., Theurillat J.-P., Raus T., Čarni A., Šumberová K., Willner W., Dengler J., Gavilán García R., Chytrý M., Hájek M., Di Pietro R., Iakushenko D., Pallas J., Daniëls F.J.A., Bergmeier E., Santos Guerra A., Ermakov N., Valachovič M., Schaminée J.H.J., Lysenko T., Didukh Y.P., Pignatti S., Rodwell J.S., Capelo J., Weber H.E., Solomeshch A., Dimopoulos P., Aguiar C., Hennekens S.M., Tichý L. (2016) Vegetation of Europe: hierarchical floristic classification system of vascular plant, bryophyte, lichen, and algal communities. *Applied Vegetation Science*, **19**, 3–264. https://doi.org/10.1111/avsc.12257

Peterka T., Hájek M., Jiroušek M., Jiménez-Alfaro B., Aunina L., Bergamini A., Dítě D., Felbaba-Klushyna L., Graf U., Hájková P., Hettenbergerová E., Ivchenko T.G., Jansen F., Koroleva N.E., Lapshina E.D., Lazarević P.M., Moen A., Napreenko M.G., Pawlikowski P., Plesková Z., Sekulová L., Smagin V.A., Tahvanainen T., Thiele A., Biţă-Nicolae C., Biurrun I., Brisse H., Ćušterevska R., De Bie E., Ewald J., FitzPatrick Ú., Font X., Jandt U., Kącki Z., Kuzemko A., Landucci F., Moeslund J.E., Pérez-Haase A., Rašomavičius V., Rodwell J.S., Schaminée J.H.J., Šilc U., Stančić Z., Chytrý

M. (2017) Formalized classification of European fen vegetation at the alliance level. *Applied Vegetation Science*, **20**, 124–142. https://doi.org/10.1111/avsc.12271

Plesková Z., Jiroušek M., Peterka T., Hájek T., Dítě D., Hájková P., Navrátilová J., Šímová A., Syrovátka V., Hájek M. (2016) Testing inter-regional variation in pH niches of fen mosses. *Journal of Vegetation Science*, **27**, 352–364. https://doi.org/10.1111/jvs.12348

QGIS.org (2026) QGIS Geographic Information System. QGIS Association, Zürich, Switzerland [software]. Available at: https://doi.org/10.5281/zenodo.6139224

R Core Team (2022). R: A language and environment for statistical computing. R Foundation for Statistical Computing, Vienna, Austria. Available at: https://www.R-project.org

Roleček J., Tichý L., Zelený D., Chytrý M. (2009) Modified TWINSPAN classification in which the hierarchy respects cluster heterogeneity. *Journal of Vegetation Science*, **20**, 596–602. https://doi.org/10.1111/j.1654-1103.2009.01062.x

Sandel B., Monnet A.C., Govaerts R., Vorontsova M. (2017) Late Quaternary climate stability and the origins and future of global grass endemism. *Annals of Botany*, **119**, 279–288. https://doi.org/10.1093/aob/mcw178

Szokala D., Kočí M., Vassilev K. (2024) Subalpine tall-herb vegetation in Bulgaria: diversity and ecology. *Plant Biosystems*, **158**, 490–510. https://doi.org/10.1080/11263504.2024.2327865

ter Braak C.J.F., Šmilauer P. (2012) Canoco reference manual and user's guide: Software for ordination, version 5.0. Microcomputer Power, Ythaca, NY.

Tichý L. (2002) JUICE, software for vegetation classification. *Journal of Vegetation Science*, **13**, 451–453. https://doi.org/10.1111/j.1654-1103.2002.tb02069.x

Tichý L., Chytrý M., Hájek M., Talbot S.S., Botta-Dukát Z. (2010b) OptimClass: Using species-to-cluster fidelity to determine the optimal partition in classification of ecological communities. *Journal of Vegetation Science*, **21**, 287–299. https://doi.org/10.1111/j.1654-1103.2009.01143.x

Tichý L., Hájek M., Zelený D. (2010a) Imputation of environmental variables for vegetation plots based on compositional similarity. *Journal of Vegetation Science*, **21**, 88–95. https://doi.org/10.1111/j.1654-1103.2009.01126.x

Tonkov S. (2021) *The postglacial vegetation history in southwestern Bulgaria*. Pensoft Publishers, Sofia, Bulgaria: 170 pp. https://doi.org/10.3897/ab.e68615

Westhoff V., van der Maarel E. (1978) The Braun-Blanquet approach. In: Whittaker R.H. (Ed), *Classification of plant communities*. Springer, Dordrecht, Netherlands: s. 287–399.

Wright H.E. Jr., Ammann B., Stefanova I., Atanassova J., Margalitadze N., Wick L., Blyakharchuk T.A. (2003) Late-glacial and early-Holocene dry climates from the Balkan Peninsula to southern Siberia. In: Tonkov S. (Ed.), *Aspects of Palynology and Palaeoecology: Festschrift in Honour of Elissaveta Bozilova*. Pensoft Publishers, Sofia, Bulgaria: 127–136.
