# Supplementary material for: Plant endemism of the Balkan high‐mountain fens: a community‐level synthesis and their climate change perspective
Source: Plant Biol (Stuttg). 2026 Aug 16;28(7):2393–409. doi: 10.1111/plb.70279 (PMC13750331; doi:10.1111/plb.70279)
Supplement: Supplementary file 3 [file PLB-28-2393-s005.pdf]

Table S4

Community richness statistics of endemics for 11 regions

**Mountain ranges grouped**	**shortening in the scatter**	**n=**	**wide-range**		**narrow-range**		**all endemics**	
			mean	*max.*	*mean*	*max.*	*mean*	*max**
Zelengora, Bjelasica, Komovi	northwest	18	2.7	5	0	0	2.7	5
Prokletje (Accursed Mts)	Prokletje (Accursed Mts)	35	3.2	6	0.5	2	3.7	7
Korab, Jablanitsa, Sharr, Kapaonik	Korab, Sharr, Jablanitsa	82	3.7	8	0.3	2	4	9
Valamara, Lurë-Dejë, Mashkullora, Smolikas, Mavrovouni	south (serpentinite)	27	1.9	4	1.7	3	3.5	6
Pelister, Varnous, Solunska glava	Pelister	11	2.6	5	1	2	3.6	6
Kaimakchalan, Vorras	Kaimakchalan	27	2.4	6	1	2	3.4	7
Vlasina plain, Osogovo	Vlasina, Osogovo	13	1.6	3	0	0	1.6	3
Rila, Pirin, Rhodopes	Rila, Pirin	98	2.9	6	1	2	3.9	8
Vitosha	Vitosha	18	4.3	7	0.4	1	4.7	7
Stara Planina	Stara Planina	29	1.8	4	0.2	1	2	5
Retezat, Făgăraș, Cindrel, Lotrului, Parâng	South Carpathians	17	2.1	4	0	0	2.1	4

*** places of the highest concentration of Balkan endemics in a relevé (for each region separately)**

Zelengora, Bjelasica, Komovi	NP Biogradska gora, Peshica (MNE)
Prokletje (Accursed Mts)	Prokletje, Treskavicka reka (MNE)
Korab, Jablanitsa, Sharr, Kapaonik	Korab Mts, NP Mavrovo, N of Crn Kamen (NMK)
Valamara, Lurë-Dejë, Mashkullora, Smolikas, Mavrovouni	Valamara, 310 m NNW of Maja Lenijes Mt (AL); Mavrovouni, 570 m E of Flenga Mt (GR)
Pelister, Varnous, Solunska glava	Pelister, Sapunchica valley (NMK)
Kaimakchalan, Vorras	Vorras, fen 1.9 km SE of Meterizi Mt (GR)
Vlasina plain, Osogovo	Vlasina plain, Bratashnica and Bukova glava fens (RS)
Rila, Pirin, Rhodopes	Rila Mts, Sedemte lakes, between the lakes Bliznaka and Bobreka (BG)
Vitosha	Vitosha Mts (between Aleko and Platoto; Torfen Rezervat) (BG)
Stara Planina	Stara Planina, 2 km S of Zltec Mt (BG)
Retezat, Făgăraș, Cindrel, Lotrului, Parâng	Retezat Mt, Zanoaga lake, Mlastina Mare (RO)
