# Supplementary material for: Plant endemism of the Balkan high‐mountain fens: a community‐level synthesis and their climate change perspective
Source: Plant Biol (Stuttg). 2026 Aug 16;28(7):2393–409. doi: 10.1111/plb.70279 (PMC13750331; doi:10.1111/plb.70279)
Supplement: Supplementary file 4 [file PLB-28-2393-s007.pdf]

Apppendix S2

Complete results of ordination analyses

The first PCA axis of abiotic factors (Fig. 5) extends from the highest eastern mountains to other areas and largely coincides with palaeoclimate and pH. The two-axis space can be interpreted well in a diagonal view, with one diagonal stretching from the east (bottom left) to the southwest (upper right); the northwest is less climatically stable, showing a larger difference between current and past minimum temperatures (LGM) and currently experiencing higher precipitation. The second diagonal follows the elevational gradients from the warm, base-rich low-elevation areas across the Balkans to the high, acidic Southern Carpathians. The paleoclimate is crucial to this gradient, because when it is not included in the analysis, the Southern Carpathians are no longer delimited from the Balkans (Fig. S4). Colder winters during glacials and a drier climate during the late pleniglacial and the Early Holocene characterise the South Carpathians against the Balkans. The richness of Balkan (sub)endemics peaks in the middle part of the second axis, where the Rila and Korab hotspots are located.

When focusing on the distribution of individual taxa along the environmental gradients (Fig. S5), *Primula frondosa* subsp. *exigua* (Velen.) O. Schwarz and *P. deorum* Velen. are restricted to the (palaeo)climatically specific part of East Balkans (Bulgaria), while *Narthecium scardicum* Košanin and *Carex castroviejoi* Luceño & Jim.Mejías are shifted to the middle part of the first (west-to-east) axis with quite a broad niche along the second axis. *Pedicularis olympica* Boiss. shows a medium position along the west-to-east gradient, but tends toward the bottom extreme (colder, wetter glacial conditions, drier summers nowadays, more southern locations). *Cirsium heterotrichum* Pančić occupies the central parts of both major gradients, tending to occur in environmentally intermediate conditions, even though they are geographically distant (e.g. the Prokletije and the Vitosha Mts). *Plantago gentianoides* Sibth. & Sm. prefers the conditions that characterise the eastern part of the study area, including the south Carpathians, although it is geographically distributed across the entire study area. On the other hand, *Gymnadenia frivaldii* Hampe ex Griseb., another wide-ranging endemic, shows a regular distribution across the two major environmental gradients.

When species composition of Balkan endemics was seen as focal and environmental variables as explanatory ones (CCA; Fig. 6), the first axis showed again the East-to-west gradient correlating with palaeoclimate, mountain massiveness and pH, while the second axis differentiated more western locations into the central Balkan area (*Pedicularis olympica*, *Crocus pelistericus* Pulević, *Epilobium vernonicum* Snogerup, *Silene asterias* Griseb.) and the more southern serpentinite areas with higher pH (*Carex castroviejoi*, *Pinguicula hirtiflora* Ten., *Narthecium scardicum*).

When focused at the level of 11 subregions (Principal Coordinate Analysis of the frequency table for Balkan endemics, Fig. S6), the first ordination axis differentiates the regions where mostly wide-ranged endemics, regionally together with *Pedicularis olympica* and *Epilobium vernonicum*, co-occur in plant communities (Kaimakchalan, Pelister, Vlasina and Stara Planina) from the regions with more narrow-ranged endemics at the community level (Korab & Šar, Prokletje, Rila & Pirin). The South Carpathians unintuitively tend to the latter group because of the shared occurrence of *Plantago gentianoides*. The second axis goes from the Korab and Šar, Prokletije and the NW part of the study area (the Dinarids) to the eastern regions.

All presented GAMs showing patterns in endemic richness along the PCA and CCA axes were statistically significant ($P < 0.001$).
