# Supplementary material for: Plant endemism of the Balkan high‐mountain fens: a community‐level synthesis and their climate change perspective
Source: Plant Biol (Stuttg). 2026 Aug 16;28(7):2393–409. doi: 10.1111/plb.70279 (PMC13750331; doi:10.1111/plb.70279)
Supplement: Supplementary file 5 [file PLB-28-2393-s010.pdf]

Table S3

Percentage synoptic table of the four major vegetation types recognised by the TWINSPAN algorithm.

Diagnostic taxa are those with the phi-coeffieint to the cluster above 0.3 and simultaneously the Fisher´s exact test significant at the P-level of 0.001. Other taxa are sorted by overall frequency in the dataset.

	frequency	cluster 1	cluster 2	cluster 3	cluster 4
**no of relevés**	*(%)*	42	103	88	143
**cluster 1**					
Pinguicula hirtiflora	*21*	48	1	0	0
Carex castroviejoi	*19*	45	0	0	0
Cratoneuron filicinum	*22*	38	6	0	0
Soldanella pindicola	*29*	38	9	0	3
Narthecium scardicum	*41*	43	12	0	8
Carex panicea	*31*	36	8	2	4
Carex kitaibeliana	*17*	26	4	1	1
Lotus corniculatus	*17*	26	4	1	1
Cirsium epiroticum	*8*	19	0	0	0
Juncus alpinoarticulatus	*117*	67	58	16	10
Eleocharis quinqueflora	*54*	43	18	11	5
Molinia caerulea agg.	*51*	43	9	25	1
Campylium stellatum	*87*	52	31	3	21
Plantago subulata s.l.	*5*	12	0	0	0
**cluster 2**					
Palustriella commutata s.l.	*96*	26	79	0	3
Cardamine acris	*62*	0	48	7	5
Carex ferruginea	*43*	5	40	0	0
Alchemilla vulgaris agg.	*113*	7	63	18	20
Willemetia stipitata subsp. *albanica*	*81*	12	52	7	11
Ptychostomum pseudotriquetrum	*183*	64	89	9	39
Palustriella decipiens	*52*	0	37	2	8
Philonotis calcarea	*40*	10	35	0	0
Heliosperma pusillum subsp. *albanicum*	*50*	17	40	0	1
Cirsium appendiculatum	*60*	7	39	11	5
Carex sempervirens	*24*	0	20	0	2
Epilobium alsinifolium	*24*	0	18	0	3
Caltha palustris	*67*	2	36	14	12
Ligusticum mutellina	*106*	7	46	6	36
**cluster 3**					
Eriophorum angustifolium	*71*	14	3	65	3
Sphagnum subsecundum	*111*	2	1	57	41
Carex rostrata	*31*	2	2	28	2
Drosera rotundifolia	*17*	0	0	19	0
Potentilla erecta	*124*	36	17	67	23
Carex echinata	*254*	19	51	88	81
Sphagnum contortum	*40*	0	6	26	8
Sphagnum squarrosum	*15*	0	1	15	1
**cluster 4**					
Straminergon stramineum	*91*	0	2	17	52
Primula frondosa subsp. *exigua*	*81*	0	12	3	46
Sphagnum platyphyllum	*54*	0	3	3	34
Juncus filiformis	*48*	0	1	3	31

no of relevés	*frequency* (%)	cluster 1 42	cluster 2 103	cluster 3 88	cluster 4 143
Scapania irrigua	*74*	0	8	8	41
Sarmentypnum exannulatum	*155*	2	15	50	66
Primula deorum	*27*	0	0	0	19
Gentiana pyrenaica	*30*	0	1	0	20
Nardus stricta	*265*	48	65	48	95
Carex lazarei	*66*	5	16	0	34
Aulacomnium palustre	*79*	2	2	24	38
Luzula sudetica	*156*	7	28	41	62
Sarmentypnum sarmentosum	*27*	0	0	3	17
Philonotis seriata	*93*	12	17	11	42
**other taxa**					
Carex nigra agg. (incl. *C. dacica*)	*304*	69	67	75	98
Pinguicula balcanica	*255*	43	71	60	78
Eriophorum latifolium	*213*	67	65	34	62
Parnassia palustris	*206*	86	74	51	34
Deschampsia cespitosa	*179*	36	68	34	45
Festuca rubra agg.	*149*	57	36	34	41
Gymnadenia frivaldii	*136*	36	35	35	38
Dactylorhiza cordigera agg.	*133*	10	50	41	29
Agrostis canina	*113*	7	5	47	45
Veratrum album agg.	*96*	21	37	26	18
Taraxacum species (*Alpina*)	*94*	31	17	6	41
Geum coccineum	*90*	10	25	22	29
Juncus articulatus	*83*	17	36	17	17
Carex flava	*80*	24	34	32	5
Plantago gentianoides	*79*	24	19	1	34
Anthoxanthum odoratum agg.	*75*	2	20	18	26
Aneura pinguis	*73*	26	29	10	16
Blysmus compressus	*72*	45	46	6	1
Scorzoneroides autumnalis	*69*	2	23	27	14
Philonotis fontana	*68*	0	33	14	15
Prunella vulgaris	*62*	12	36	15	5
Eriophorum vaginatum	*60*	0	0	23	28
Sphagnum teres	*59*	0	7	25	21
Trifolium pratense	*57*	21	25	14	7
Saxifraga stellaris subsp. *engleri*	*56*	5	17	3	23
Epilobium palustre	*56*	0	19	27	8
Cardamine rivularis	*53*	0	5	11	27
Carex viridula	*52*	12	22	13	9
Bruckenthalia spiculifolia	*49*	5	9	13	19
Vaccinium uliginosum	*49*	0	9	10	21
Scorpidium revolvens agg. (*revolvens* , *cossonii*)	*46*	24	24	1	7
Sesleria comosa	*44*	0	5	9	22
Selaginella selaginoides	*43*	21	21	0	8
Trifolium badium	*42*	5	26	7	5
Gentianella bulgarica	*41*	12	12	13	9
Agrostis stolonifera agg.	*40*	19	18	5	6
Carex canescens	*38*	0	3	20	12
Calliergonella cuspidata	*37*	7	17	10	5
Sanguisorba officinalis	*36*	14	6	22	3

no of relevés	*frequency* (%)	**cluster 1** 42	**cluster 2** 103	**cluster 3** 88	**cluster 4** 143
Myosotis palustris agg.	*36*	0	6	14	13
Sphagnum capillifolium	*36*	0	0	16	15
Equisetum palustre	*35*	17	13	14	2
Riccardia multifida	*35*	24	11	3	8
Epilobium nutans	*34*	0	7	1	18
Juncus thomasii	*34*	0	15	14	5
Cirsium heterotrichum	*33*	14	7	15	5
Scorzoneroides rilaensis	*33*	0	5	3	17
Trifolium hybridum	*32*	7	12	9	6
Succisa pratensis	*31*	10	3	25	1
Ranunculus sartorianus	*31*	24	7	5	7
Pedicularis olympica (limnogena)	*31*	0	4	11	12
Vaccinium myrtillus	*30*	2	5	3	15
Polytrichum commune	*30*	0	1	11	13
Climacium dendroides	*29*	2	8	14	6
Carex davalliana	*29*	26	17	0	0
Linum catharticum	*29*	17	17	2	1
Allium schoenoprasum	*29*	2	16	6	5
Chiloscyphus polyanthos agg.	*29*	0	11	5	10
Cynosurus cristatus	*28*	5	17	8	1
Galium palustre	*27*	2	6	20	1
Trichophorum cespitosum	*27*	0	2	9	12
Dichodon cerastoides	*27*	0	4	2	15
Pellia neesiana	*26*	0	13	1	8
Sphagnum warnstorfii	*25*	0	0	13	10
Juncus effusus	*24*	0	9	15	1
Carex lepidocarpa	*23*	14	16	1	0
Carex paniculata	*23*	7	19	0	0
Soldanella chrysosticta	*23*	0	6	1	11
Scapania undulata	*22*	0	6	1	10
Luzula campestris agg.	*22*	0	7	11	3
Ranunculus acris	*21*	10	4	14	1
Angelica sylvestris agg.	*21*	2	10	8	2
Diobelonella palustris	*20*	0	7	1	8
Trifolium repens s.l.	*20*	0	12	2	4
Briza media	*19*	12	8	7	0
Hamatocaulis vernicosus	*19*	5	0	13	4
Brachythecium rivulare	*19*	2	13	1	3
Juniperus communis subsp. *nana*	*18*	2	3	5	7
Sphagnum russowii	*18*	0	0	3	10
Dicranum bonjeanii	*18*	0	0	3	10
Agrostis capillaris	*17*	2	6	6	3
Homogyne alpina	*17*	0	3	0	10
Utricularia minor	*16*	7	1	0	8
Crocus veluchensis	*16*	0	1	9	5
Vaccinium vitis-idaea	*16*	0	1	13	3
Cerastium fontanum subsp. *vulgare*	*16*	0	13	1	1
Ranunculus polyanthemos subsp. *nemorosus*	*15*	7	2	2	6
Leontodon hispidus	*15*	5	11	1	1
Euphrasia minima	*15*	2	12	0	1

no of relevés	*frequency* (%)	**cluster 1** 42	**cluster 2** 103	**cluster 3** 88	**cluster 4** 143
Blindia acuta	*14*	0	8	0	4
Epilobium vernonicum	*14*	0	4	8	2
Crocus pelistericus	*14*	0	1	7	5
Pinus peuce	*13*	2	5	1	4
Valeriana dioica subsp. *simplicifolia*	*13*	2	4	8	1
Ptychostomum schleicheri	*13*	5	7	1	2
Viola palustris	*13*	0	1	7	4
Sagina procumbens	*12*	0	7	2	2
Silene asterias	*12*	0	7	5	1
Crepis paludosa	*12*	0	3	8	1
Calliergonella lindbergii	*12*	0	3	3	4
Avenella flexuosa	*12*	0	0	2	7
Danthonia decumbens	*11*	14	2	1	1
Carex umbrosa	*11*	7	4	1	2
Bistorta officinalis	*11*	2	4	7	0
Geum rivale	*11*	0	6	3	1
Geranium sylvaticum	*11*	0	6	1	3
Sphagnum compactum	*11*	0	0	0	8
Lathyrus pratensis	*10*	2	4	5	1
Plantago lanceolata	*10*	10	5	1	0
Soldanella alpina	*10*	7	6	1	0
Ranunculus montanus aggr.	*10*	0	4	3	2
Equisetum fluviatile	*10*	0	1	9	1
Juncus conglomeratus	*10*	0	0	11	0
Campanula patula	*10*	0	1	9	1
Mentha longifolia	*9*	5	5	2	0
Fissidens adianthoides	*9*	12	4	0	0
Euphrasia species	*9*	5	4	0	2
Juncus triglumis	*9*	5	6	0	1
Hypericum maculatum	*9*	0	5	0	3
Rhizomnium punctatum agg.	*9*	0	5	0	3
Pinus mugo	*9*	0	1	0	6
Carex leporina	*9*	0	7	2	0
Epilobium anagallidifolium	*9*	0	4	2	2
Cephalozia bicuspidata	*9*	0	1	0	6
Trifolium spadiceum	*9*	0	3	7	0
Scirpus sylvaticus	*9*	0	2	6	1
Scapania paludicola	*9*	0	0	0	6
Equisetum arvense	*8*	5	5	1	0
Luzula luzulina	*8*	5	1	2	2
Dianthus superbus	*8*	5	1	6	0
Phleum alpinum	*8*	0	3	2	2
Saxifraga aizoides	*8*	0	8	0	0
Plagiomnium ellipticum	*8*	0	4	3	1
Picea abies	*8*	0	3	3	1
Pseudorchis albida	*8*	0	0	7	1
Philonotis tomentella	*7*	2	4	0	1
Holcus lanatus	*7*	5	1	5	0
Primula halleri	*7*	10	3	0	0
Plagiomnium elatum	*7*	2	4	2	0

no of relevés	*frequency* (%)	**cluster 1** 42	**cluster 2** 103	**cluster 3** 88	**cluster 4** 143
Rhinanthus minor	*7*	2	2	3	1
Menyanthes trifoliata	*7*	5	0	5	1
Luzula alpinopilosa	*7*	0	2	0	3
Bistorta vivipara	*7*	0	6	1	0
Polytrichum formosum	*7*	0	1	2	3
Veronica beccabunga	*7*	0	7	0	0
Rumex alpinus	*7*	0	5	0	1
Sphagnum magellanicum	*7*	0	0	7	1
Salix lapponum	*7*	0	0	6	1
Calliergon giganteum	*7*	0	2	2	2
Sphagnum flexuosum	*7*	0	0	6	1
Sphagnum centrale	*7*	0	0	5	2
Pohlia nutans	*6*	2	0	0	3
Euphrasia salisburgensis	*6*	2	4	0	1
Carex flacca	*6*	12	1	0	0
Veronica serpyllifolia s.l.	*6*	2	3	1	1
Triglochin palustris	*6*	2	2	1	1
Saxifraga rotundifolia	*6*	0	6	0	0
Poa alpina	*6*	0	5	0	1
Poa media	*6*	0	1	2	2
Anthoxanthum alpinum	*6*	0	1	0	3
Phyteuma pseudorbiculare	*6*	0	5	0	1
Bartsia alpina	*6*	0	3	0	2
Ochlopoa annua	*6*	0	3	0	2
Carex demissa	*6*	0	6	0	0
Rumex balcanicus	*6*	0	3	2	1
Jacobaea subalpina	*6*	0	1	1	3
Alnus viridis	*6*	0	1	6	0
Carex hirta	*6*	0	6	0	0
Atrichum undulatum	*6*	0	0	6	1
Eleocharis uniglumis	*5*	10	1	0	0
Gentiana asclepiadea	*5*	2	1	3	0
Tomentypnum nitens	*5*	2	0	3	1
Euphrasia rostkoviana	*5*	5	1	2	0
Carex lasiocarpa	*5*	5	0	3	0
Cetraria islandica	*5*	2	0	0	3
Festuca species	*5*	5	2	0	1
Mesoptychia bantriensis	*5*	2	3	0	1
Tussilago farfara	*5*	2	4	0	0
Euphrasia stricta	*5*	0	4	0	1
Jacobaea pancicii	*5*	0	1	0	3
Marchantia polymorpha	*5*	0	4	0	1
Salix herbacea	*5*	0	1	1	2
Gentiana verna subsp. balcanica	*5*	0	5	0	0
Schedonorus pratensis	*5*	0	4	1	0
Stellaria alsine	*5*	0	3	1	1
Luzula luzuloides agg.	*5*	0	1	0	3
Ranunculus repens	*5*	0	5	0	0
Sphagnum inundatum	*5*	0	0	2	2
Sphagnum fallax	*5*	0	0	3	1

no of relevés	*frequency* (%)	**cluster 1** 42	**cluster 2** 103	**cluster 3** 88	**cluster 4** 143
Polytrichum strictum	*5*	0	0	2	2
Sphagnum angustifolium	*5*	0	0	3	1
Pedicularis palustris	*5*	0	0	6	0
Comarum palustre	*5*	0	0	6	0
Carex pauciflora	*5*	0	0	0	3
Juncus inflexus	*4*	7	1	0	0
Trollius europaeus	*4*	2	2	0	1
Epilobium parviflorum	*4*	10	0	0	0
Cardamine matthioli	*4*	2	1	2	0
Carex buxbaumii	*4*	7	1	0	0
Eleocharis palustris	*4*	7	1	0	0
Eleocharis acicularis	*4*	5	0	2	0
Fissidens osmundoides	*4*	0	4	0	0
Agrostis rupestris	*4*	0	1	0	2
Ceratodon purpureus	*4*	0	2	0	1
Salix waldsteiniana	*4*	0	3	1	0
Pedicularis verticillata	*4*	0	4	0	0
Gymnadenia conopsea	*4*	0	3	0	1
Chaerophyllum hirsutum	*4*	0	3	0	1
Cardamine pratensis	*4*	0	0	5	0
Sphagnum girgensohnii	*4*	0	0	1	2
Epilobium obscurum	*4*	0	1	2	1
Scutellaria galericulata	*4*	0	0	5	0
Sphagnum palustre	*4*	0	0	1	2
Marsupella sphacelata	*4*	0	0	0	3
Epipactis palustris	*3*	7	0	0	0
Carex distans	*3*	7	0	0	0
Ononis spinosa subsp. *hircina*	*3*	7	0	0	0
Tortella tortuosa	*3*	5	1	0	0
Ajuga reptans	*3*	5	1	0	0
Dactylorhiza incarnata	*3*	5	1	0	0
Silene flos-cuculi	*3*	2	0	2	0
Polygala vulgaris	*3*	5	0	1	0
Dactylorhiza maculata agg.	*3*	2	2	0	0
Salix caprea	*3*	2	1	1	0
Gentiana utriculosa	*3*	2	0	1	1
Pinus heldreichii	*3*	7	0	0	0
Philonotis caespitosa	*3*	0	1	0	1
Ranunculus bulbosus	*3*	0	1	0	1
Sagina saginoides	*3*	0	2	0	1
Cardamine amara	*3*	0	3	0	0
Potentilla crantzii	*3*	0	1	0	1
Crepis tectorum	*3*	0	3	0	0
Ptilidium ciliare	*3*	0	1	0	1
Thalictrum alpinum	*3*	0	3	0	0
Ranunculus degenii	*3*	0	3	0	0
Glyceria notata	*3*	0	3	0	0
Pohlia wahlenbergii	*3*	0	3	0	0
Ptychostomum pallens	*3*	0	1	0	1
Poa trivialis	*3*	0	2	1	0

no of relevés	*frequency* (%)	**cluster 1** 42	**cluster 2** 103	**cluster 3** 88	**cluster 4** 143
Poa palustris	*3*	0	2	1	0
Ranunculus breyninus	*3*	0	3	0	0
Trifolium species	*3*	0	2	0	1
Sphagnum rubellum	*3*	0	0	3	0
Melampyrum scardicum	*3*	0	0	2	1
Senecio nemorensis agg.	*3*	0	0	1	1
Polytrichum longisetum	*3*	0	0	3	0
Betula pubescens	*3*	0	0	3	0
Empetrum nigrum	*3*	0	0	0	2
Marsupella emarginata	*3*	0	0	0	2
Jasione laevis	*3*	0	0	0	2
Cladonia species	*3*	0	0	0	2
Polytrichum juniperinum	*3*	0	0	0	2
Carex acutiformis	*2*	5	0	0	0
Mentha x verticillata	*2*	5	0	0	0
Cirsium ligulare	*2*	5	0	0	0
Lythrum salicaria	*2*	2	0	1	0
Carduus tmoleus	*2*	5	0	0	0
Ranunculus polyanthemos	*2*	2	1	0	0
Carex hostiana	*2*	5	0	0	0
Drepanocladus trifarius	*2*	5	0	0	0
Crepis species	*2*	2	1	0	0
Hieracium species	*2*	2	1	0	0
Phragmites australis	*2*	5	0	0	0
Juncus species	*2*	2	1	0	0
Danthonia alpina	*2*	2	1	0	0
Pinus sylvestris	*2*	2	0	1	0
Poa cenisia	*2*	0	2	0	0
Sesleria caerulea	*2*	0	1	0	1
Ligusticum albanicum	*2*	0	2	0	0
Jungermannia species	*2*	0	2	0	0
Pedicularis species	*2*	0	2	0	0
Gentiana acaulis	*2*	0	1	1	0
Geum montanum	*2*	0	1	0	1
Plantago media	*2*	0	2	0	0
Juncus anceps subsp. *herzegovinus*	*2*	0	1	0	1
Galium album agg.	*2*	0	1	1	0
Dichodontium pellucidum	*2*	0	2	0	0
Doronicum austriacum	*2*	0	2	0	0
Poa species	*2*	0	2	0	0
Rumex acetosa agg.	*2*	0	1	1	0
Ptychostomum pallescens	*2*	0	1	0	1
Epilobium species	*2*	0	2	0	0
Viola biflora	*2*	0	1	0	1
Ranunculus carinthiacus	*2*	0	1	1	0
Cruciata glabra	*2*	0	1	0	1
Sanionia uncinata	*2*	0	1	0	1
Ptychostomum weigelii	*2*	0	1	0	1
Rhinanthus rumelicus	*2*	0	2	0	0
Ranunculus flammula	*2*	0	1	1	0

no of relevés	*frequency* (%)	**cluster 1** 42	**cluster 2** 103	**cluster 3** 88	**cluster 4** 143
Plantago major	*2*	0	2	0	0
Carex caryophyllea	*2*	0	1	1	0
Poa pratensis	*2*	0	1	1	0
Luzula congesta	*2*	0	1	0	1
Carex species	*2*	0	2	0	0
Dactylorhiza viridis	*2*	0	2	0	0
Brachythecium mildeanum	*2*	0	1	1	0
Galium species	*2*	0	2	0	0
Ptychostomum inclinatum	*2*	0	1	0	1
Dactylorhiza species	*2*	0	1	1	0
Cirsium species	*2*	0	1	0	1
Sphagnum subnitens	*2*	0	0	2	0
Rubus idaeus	*2*	0	0	2	0
Sphagnum auriculatum	*2*	0	0	2	0
Luzula sylvatica	*2*	0	0	2	0
Crepis biennis	*2*	0	0	2	0
Leucanthemum graminifolium	*2*	0	0	2	0
Dianthus barbatus	*2*	0	0	2	0
Salix aurita	*2*	0	0	2	0
Carex limosa	*2*	0	0	2	0
Juncus acutiflorus	*2*	0	0	1	1
Pyrola rotundifolia	*2*	0	0	2	0
Geum rhodopeum	*2*	0	0	2	0
Filipendula ulmaria	*2*	0	0	2	0
Primula veris	*2*	0	0	2	0
Centaurea jacea	*2*	0	0	2	0
Vicia cracca	*2*	0	0	2	0
Dactylorhiza baumanniana	*2*	0	0	2	0
Utricularia ochroleuca	*2*	0	0	1	1
Calypogeia sphagnicola	*2*	0	0	0	1
Cephalozia species	*2*	0	0	0	1
Alopecurus riloensis	*2*	0	0	0	1
Drepanocladus aduncus	*2*	0	0	0	1
Pinguicula vulgaris	*2*	0	0	0	1
Calypogeia azurea	*2*	0	0	0	1
Cynodontium strumiferum	*2*	0	0	0	1
Sesleria coerulans	*2*	0	2	0	0
Potentilla reptans	*1*	2	0	0	0
Harpanthus flotovianus	*1*	2	0	0	0
Antennaria dioica	*1*	2	0	0	0
Hypnum cupressiforme	*1*	2	0	0	0
Carlina acaulis	*1*	2	0	0	0
Populus tremula	*1*	2	0	0	0
Vicia cracca subsp. incana	*1*	2	0	0	0
Erica carnea	*1*	2	0	0	0
Gentianella crispata	*1*	2	0	0	0
Fagus sylvatica	*1*	2	0	0	0
Crocus species	*1*	2	0	0	0
Salvia officinalis	*1*	2	0	0	0
Sesleria species	*1*	2	0	0	0

no of relevés	*frequency* *(%)*	**cluster 1** 42	**cluster 2** 103	**cluster 3** 88	**cluster 4** 143
Equisetum variegatum	*1*	2	0	0	0
Mentha arvensis	*1*	2	0	0	0
Polygala amarella	*1*	2	0	0	0
Hypericum tetrapterum	*1*	2	0	0	0
Trifolium parnassi	*1*	2	0	0	0
Gentianella species	*1*	2	0	0	0
Dicranum leioneuron	*1*	2	0	0	0
Racomitrium elongatum	*1*	2	0	0	0
Crocus albanicus	*1*	2	0	0	0
Thesium species	*1*	2	0	0	0
Daphne oleoides	*1*	2	0	0	0
Thymus pulegioides	*1*	2	0	0	0
Peucedanum species	*1*	2	0	0	0
Equisetum x moorei	*1*	2	0	0	0
Equisetum ramosissimum	*1*	2	0	0	0
Pedicularis comosa	*1*	2	0	0	0
Polytrichum species	*1*	2	0	0	0
Chara species	*1*	2	0	0	0
Juncus atratus	*1*	2	0	0	0
Riccardia species	*1*	2	0	0	0
Didymodon luridus	*1*	0	1	0	0
Philonotis marchica	*1*	0	1	0	0
Mesoptychia badensis	*1*	0	1	0	0
Marchantia quadrata	*1*	0	1	0	0
Cynodontium species	*1*	0	1	0	0
Hypericum richeri s.l.	*1*	0	1	0	0
Thymus praecox subsp. *jankae*	*1*	0	1	0	0
Bellis perennis	*1*	0	1	0	0
Gentiana punctata	*1*	0	1	0	0
Dicranum scoparium	*1*	0	1	0	0
Phyteuma species	*1*	0	1	0	0
Luzula forsteri	*1*	0	1	0	0
Trisetum alpestre	*1*	0	1	0	0
Montia fontana	*1*	0	1	0	0
Salix species	*1*	0	1	0	0
Dicranum polysetum	*1*	0	1	0	0
Lysimachia nummularia	*1*	0	1	0	0
Primula elatior	*1*	0	1	0	0
Centaurium erythraea	*1*	0	1	0	0
Imbribryum alpinum	*1*	0	1	0	0
Hieracium speciosum	*1*	0	1	0	0
Viola canina	*1*	0	1	0	0
Rhinanthus species	*1*	0	1	0	0
Salix amplexicaulis	*1*	0	1	0	0
Plagiochila porelloides	*1*	0	1	0	0
Pilosella piloselloides	*1*	0	1	0	0
Gymnostomum aeruginosum	*1*	0	1	0	0
Pilosella lactucella	*1*	0	1	0	0
Saxifraga ferdinandi-coburgi	*1*	0	1	0	0
Amblyodon dealbatus	*1*	0	1	0	0

no of relevés	*frequency* (%)	**cluster 1** 42	**cluster 2** 103	**cluster 3** 88	**cluster 4** 143
Onobrychis montana subsp. *scardica*	*1*	0	1	0	0
Euphrasia pectinata	*1*	0	1	0	0
Oenanthe silaifolia	*1*	0	1	0	0
Solenostoma gracillimum	*1*	0	1	0	0
Scapania curta	*1*	0	1	0	0
Petasites albus	*1*	0	1	0	0
Galium anisophyllon	*1*	0	1	0	0
Acer pseudoplatanus	*1*	0	1	0	0
Sesleria uliginosa	*1*	0	1	0	0
Trifolium pallescens	*1*	0	1	0	0
Polygala alpestris subsp. *croatica*	*1*	0	1	0	0
Hypericum richeri subsp. *grisebachii*	*1*	0	1	0	0
Solenostoma obovatum	*1*	0	1	0	0
Eriophorum gracile	*1*	0	1	0	0
Thymus species	*1*	0	1	0	0
Achillea ptarmica	*1*	0	1	0	0
Dicranum spadiceum	*1*	0	1	0	0
Poa badensis	*1*	0	1	0	0
Cystopteris fragilis	*1*	0	1	0	0
Viola suavis	*1*	0	1	0	0
Cirsium canum	*1*	0	1	0	0
Galanthus species	*1*	0	1	0	0
Epilobium gemmascens	*1*	0	1	0	0
Clinopodium vulgare	*1*	0	1	0	0
Rumex obtusifolius subsp. transiens	*1*	0	1	0	0
Plagiomnium affine	*1*	0	1	0	0
Meesia triquetra	*1*	0	1	0	0
Cardamine species	*1*	0	1	0	0
Sagina species	*1*	0	1	0	0
Brachypodium pinnatum	*1*	0	1	0	0
Agrostis species	*1*	0	1	0	0
Sisyrinchium montanum	*1*	0	1	0	0
Hypericum perforatum	*1*	0	1	0	0
Salix cinerea	*1*	0	1	0	0
Athyrium distentifolium	*1*	0	1	0	0
Veronica urticifolia	*1*	0	1	0	0
Fragaria vesca	*1*	0	1	0	0
Phyteuma orbiculare	*1*	0	1	0	0
Cerastium alpinum	*1*	0	1	0	0
Swertia perennis	*1*	0	1	0	0
Armeria canescens subsp. *nebrodensis*	*1*	0	1	0	0
Hypericum species	*1*	0	1	0	0
Leucanthemum vulgare	*1*	0	1	0	0
Trifolium medium	*1*	0	0	1	0
Sphagnum papillosum	*1*	0	0	1	0
Calamagrostis villosa	*1*	0	0	1	0
Rumex species	*1*	0	0	1	0
Danthonia decumbens	*1*	0	0	1	0
Pilosella caespitosa	*1*	0	0	1	0
Scabiosa columbaria	*1*	0	0	1	0

no of relevés	*frequency* (%)	cluster 1 42	cluster 2 103	cluster 3 88	cluster 4 143
Achillea millefolium agg.	*1*	0	0	1	0
Epilobium montanum	*1*	0	0	1	0
Senecio species	*1*	0	0	1	0
Carex brizoides	*1*	0	0	1	0
Sphagnum cuspidatum	*1*	0	0	1	0
Vaccinium oxycoccos	*1*	0	0	1	0
Lysimachia vulgaris	*1*	0	0	1	0
Veronica scutellata	*1*	0	0	1	0
Carex acuta	*1*	0	0	1	0
Genista species	*1*	0	0	1	0
Ranunculus serbicus	*1*	0	0	1	0
Thalictrum aquilegiifolium	*1*	0	0	1	0
Rhytidiadelphus squarrosus	*1*	0	0	1	0
Carex vesicaria	*1*	0	0	1	0
Koeleria macrantha	*1*	0	0	1	0
Festuca stricta subsp. *sulcata*	*1*	0	0	1	0
Trifolium velenovskyi	*1*	0	0	1	0
Pseudohygrohypnum eugyrium	*1*	0	0	1	0
Gentiana albanica	*1*	0	0	1	0
Chondrilla latifolia	*1*	0	0	1	0
Oenanthe banatica	*1*	0	0	1	0
Holcus mollis	*1*	0	0	1	0
Calliergon cordifolium	*1*	0	0	1	0
Cirsium palustre	*1*	0	0	1	0
Patzkea paniculata	*1*	0	0	0	1
Helictochloa versicolor	*1*	0	0	0	1
Pinus nigra	*1*	0	0	0	1
Agrostis alpina	*1*	0	0	0	1
Gymnocolea inflata	*1*	0	0	0	1
Campanula alpina	*1*	0	0	0	1
Polytrichum piliferum	*1*	0	0	0	1
Gnaphalium supinum	*1*	0	0	0	1
Soldanella pusilla	*1*	0	0	0	1
Mylia anomala	*1*	0	0	0	1
Fuscocephaloziopsis pleniceps	*1*	0	0	0	1
Soldanella hungarica	*1*	0	0	0	1
Aconitum tauricum	*1*	0	0	0	1
Juncus effusus	*1*	0	0	0	1
Sphagnum molle	*1*	0	0	0	1
Vicia species	*1*	0	0	0	1
Timmia austriaca	*1*	0	0	0	1
Hygroamblystegium humile	*1*	0	0	0	1
Euphrasia liburnica	*1*	0	0	0	1
Scorpidium scorpioides	*1*	0	0	0	1
Polytrichastrum alpinum	*1*	0	0	0	1
Scapania subalpina	*1*	0	0	0	1
Meesia longiseta	*1*	0	0	0	1
Hygrohypnum luridum	*1*	0	0	0	1
Veronica bellidioides	*1*	0	0	0	1
Scapania species	*1*	0	0	0	1

no of relevés	*frequency* (%)	cluster 1 42	cluster 2 103	cluster 3 88	cluster 4 143
Solenostoma hyalinum	*1*	0	0	0	1
Racomitrium aquaticum	*1*	0	0	0	1
Racomitrium microcarpon	*1*	0	0	0	1
Tayloria lingulata	*1*	0	0	0	1
Primula minima	*1*	0	0	0	1
Scapania mucronata	*1*	0	0	0	1
Nardia scalaris	*1*	0	0	0	1
Meesia uliginosa	*1*	0	0	0	1
Paludella squarrosa	*1*	0	0	0	1
Galium uliginosum	*1*	0	0	0	1
Racomitrium sudeticum	*1*	0	0	0	1
