# Supplementary material for: Plant endemism of the Balkan high‐mountain fens: a community‐level synthesis and their climate change perspective
Source: Plant Biol (Stuttg). 2026 Aug 16;28(7):2393–409. doi: 10.1111/plb.70279 (PMC13750331; doi:10.1111/plb.70279)
Supplement: Supplementary file 6 [file PLB-28-2393-s012.pdf]

Table S2. Data sources

The author´s abbreviations refer to the authors of this study; other authors are mentioned by full name. The leading authors are in bold. Many other researchers contributed to the sampling of some relevés: Martin Kočí, Kateřina Kočí, Petr Wolf, Zuzana Fajmonová, Tenyo Meshinev, Branislav Kopčan, Alexandra Smolková, Kristýna Ratajová, Natálie Hoňková, Jiří Honěk, Jitka Wolfová, Rossen Tsonev, Nikolay Velev.

* = one plot was removed from the Spearman´s correlations, SARs, PCA and CCA as wrong coordinates were revealed during the data processing, keeping 35 plots from this subset.

**Region**	**Authors/Source**	**Plots**	**Plots with mosses**	**References**
Bulgaria	**MH, PH**, **IA**, DSop	135	135	Hájková *et al.* 2006 (only synoptic table and analyses), partially Hájek *et al.* 2021
West and South Balkans	**MH, PH, DD,** ZP, MCh, DM, LS, RČ, SH, IA, DSop	89	89	unpublished data
Šar Mts (N Macedonia)	Lupcho Melovski	36*	36*	unpublished data (deceased author)
Greece	**EB**	32	20	unpublished data
Montenegro, N Macedonia	**TP**	4	4	unpublished data
Serbia, Kosovo	European Mire Vegetation Database (EMVD)	32	32	Ranđelović & Zlatković (2010), Ranđelović *et al.* 1998, Čolić 1965
Kosovo	**NB**	23	4	Berisha 2023 and unpublished data
Greece (Smolikas Mts)	Mário Duchoň	4	3	Duchoň 2018
Serbia, Kosovo	**PL**	19	18	Lazarević 2016 (relevés available in EMVD)
Montenegro	**DSzok, BV**	9	8	unpublished data
Bulgaria, Montenegro	**DSzok**	10	10	Szokala 2023 and unpublished data
South Carpathians	**MH, PH, IG**	17	17	unpublished data, partially Hájek *et al.* 2021

Author abbreviations

MH = Michal Hájek, PH = Petra Hájková, IA = Iva Apostolova, DSop = Desislava Sopotlieva, DD = Daniel Dítě, ZP = Zuzana Plesková, MCh = Marija Chobanova (Trencheva), DM = Đorđije Milanović, LS = Lulëzim Shuka, RČ = Renata Čusterevska, SH = Slavcho Hristovski, EB = Erwin Bergmeier, TP = Tomáš Peterka, NB = Naim Berisha, PL = Predrag Lazarević, DSzok = Daniel Szokala, BV = Branislav Vrábel, IG = Irina Goia

References

Berisha N., Ćušterevska R., Millaku F., Matevski V. (2023) *Blysmo compressi-Eriophoretum latifoliae* ass. nova, a new association of the *Caricion fuscae* alliance from the Sharri Mountains. *Plant Sociology*, **60**, 37–49. https://doi.org/10.3897/pls2023601/04

Čolić D. (1965) Nova nalazišta rosulje (*Drosera rotundifolia* L.) na Staroj planini – istočna Srbija [New findings of common sundew (*Drosera rotundifolia* L.) on Stara planina Mt. – Eastern Serbia]. *Zaštita Prirode*, **29–30**, 5–23.

Duchoň M. (2018) Alpine vegetation of Mount Smolikas (Greece) and Mount Nemërçkë (Albania) [Master thesis]. Masaryk University, Brno, Czech Republic. https://doi.org/10.13140/RG.2.2.11978.52166

Hájek M., Hájková P., Apostolova I., Sopotlieva D., Goia I., Dítě D. (2021) The vegetation of rich fens (*Sphagno warnstorfii-Tomentypnion nitentis*) at the southeastern margins of their European range. *Vegetation Classification and Survey*, **2**, 177–190. https://doi.org/10.3897/VCS/2021/69118

Hájková P., Hájek M., Apostolova I. (2006) Diversity of wetland vegetation in the Bulgarian high mountains, main gradients and context-dependence of the pH role. *Plant Ecology*, **184**, 111–130. https://doi.org/10.1007/s11258-005-9056-5

Lazarević P. (2016) Tresave Srbije klase *Scheuchzerio-Caricetea fuscae* (Nordh. 1936) R. Tx. 1937: florističko-vegetacijske karakteristike, ugroženost i zaštita [Bogs of Serbia of the class Scheuchzerio-Caricetea fuscae (Nordh. 1936) R. Tx. 1937 - floristic-vegetation characteristics, endangerment and protection] [PhD thesis]. Biološki fakultet, Univerzitet u Beogradu, Belgrade, Serbia.

Ranđelović V.N., Zlatković B.K. (2010) *Flora i vegetacija Vlasinske visoravni*. Univerzitet u Nišu, Niš, Serbia.

Ranđelović V.N., Zlatković B.K., Amiđić L. (1998) Flora and vegetation of high-mountain peat-bogs of Mt. Šar-planina. *University Thought*, **5**, 91–98.

Szokala D. (2023) Alpine and subalpine acidophilous vegetation on the eastern side of the Chiprovska Planina Mts. *Tuexenia*, **43**, 109–158. https://doi.org/10.14471/2023.43.001
